# scRNA-seq Can Identify Different Cell Populations in Ovarian Cancer Bulk RNA-seq Experiments

**DOI:** 10.3390/ijms26157512

**Published:** 2025-08-04

**Authors:** Sofia Gabrilovich, Eric Devor, Nicholas Cardillo, David Bender, Michael Goodheart, Jesus Gonzalez-Bosquet

**Affiliations:** 1 Department of Obstetrics and Gynecology, Medical College of Wisconsin, 9200 W Wisconsin Ave, Milwaukee, WI 53226, USA; 2 Department of Obstetrics and Gynecology, University of Iowa, 200 Hawkins Dr., Iowa City, IA 52242, USA; eric-devor@uiowa.edu (E.D.); david-bender@uiowa.edu (D.B.); michael-goodheart@uiowa.edu (M.G.); 3 Department of Obstetrics and Gynecology, Jefferson Health, 3941 Commerce Ave, Willow Grove, PA 19090, USA

**Keywords:** ovarian cancer, genetic variation, RNA sequencing, single-cell RNA sequencing

## Abstract

High-grade serous ovarian cancer (HGSC) is a heterogeneous disease. RNA sequencing (RNAseq) of bulk solid tissue is of limited use in these populations due to heterogeneity. Single-cell RNA-seq (scRNA-seq) allows for the identification of diverse genetic compositions of heterogeneous cell populations. New computational methodologies are now available that use scRNAseq results to estimate cell type proportions in bulk RNAseq data. We performed bulk RNA-seq gene expression analysis on 112 HGSC specimens and 12 benign fallopian tube (FT) controls. We identified several publicly available scRNAseq datasets for use as annotation and reference datasets. Deconvolution was performed with MUlti-Subject SIngle Cell Deconvolution (MuSiC) to estimate cell type proportions in the bulk RNA-seq data. Datasets from the Cancer Genome Atlas (TCGA). HGSC repositories were also evaluated. Clinical variables and percentages of cell types were compared for differences in clinical outcomes and treatment results. Pathway enrichment analysis was also performed. Different annotations for referenced scRNA-seq datasets used for deconvolution of bulk RNA-seq data revealed different cellular proportions that were significantly associated with clinical outcomes; for example, higher proportions of macrophages were associated with a better response to primary chemotherapy. Our deconvolution study of bulk RNAseq HGSC samples identified cell populations within the tumor that may be associated with some of the observed clinical outcomes.

## 1. Introduction

Ovarian cancer is a heterogeneous disease. Even the most common type of ovarian cancer, high-grade serous ovarian cancer (HGSC), has significant genetic heterogeneity between and within tumors [1,2]. Consequently, not all patients respond to current standard therapies, which include surgical cytoreduction and platinum-based doublet chemotherapies with or without maintenance therapy for targeted patients [3,4]. Indeed, between 25% and 30% of HGSC patients will not respond to initial treatment [5], and almost 80% of stage III and IV patients will experience recurrence despite an initial clinical complete response [6]. Despite extensive efforts to predict which patients will respond to this initial treatment [5,7,8,9,10], more research is needed to understand the heterogeneity of the disease and its response to therapy. Understanding the diverse molecular make-up of ovarian cancer and its association with treatment outcome may inform novel alternative treatment strategies that could be used for patients who will not respond initially.

RNA sequencing (RNAseq) of bulk tissue or bulk solid tumors measures average gene expression in tissue specimens. HGSC tumors have multiple cell populations, including noncancer cells, and each population may be in different states, so-called cancer cell plasticity. The molecular characterization of ovarian cancer has become a contentious topic in the field of gynecologic oncology in the last decade, with multiple attempts at subtype classification. Tothill et al. described six clinically relevant subtypes of ovarian malignancies, with four of these subtypes representing serous and endometrioid morphology that was higher grade and advanced stage [11]. The TCGA also included four subtypes but did not describe patient survival differences [12]. A separate de novo molecular classification of the TCGA database by Konecny et al. revealed the prognostic relevance of four subtypes: mesenchymal, proliferative, immunoreactive and differentiated [13]. Ultimately, consensusOV was published that combined three major subtyping methods and proposed a standardized classification for only the most unambiguously classifiable tumors [14]. These efforts further support the need for additional understanding of the heterogeneity of ovarian cancer. This heterogeneity cannot be addressed by bulk RNAseq only. Single-cell RNA-seq (scRNA-seq) determines gene expression in a single cell, allowing for the identification of diverse genetic make-ups of diverse cell populations [15]. Limitations of scRNA-seq include cost and sample preparation steps that destroy certain cell types, making it ill-suited for use with solid tissues [15,16]. New computational methodologies are now available that use scRNAseq results to estimate cell type proportions in bulk RNAseq data. This process is termed deconvolution [17].

There is mounting evidence that HGSC originates from the fallopian tube epithelium. [18,19,20] Additionally, scRNA-seq of normal epithelial cells from the fallopian tube has been used to identify genomic heterogeneity in HGSC. Data from scRNA-seq of the normal tubal epithelium could be used to deconvolute bulk RNA-seq data from datasets integrated with clinical outcome data to identify cells that are associated with worse prognoses. ScRNAseq data have been used to identify immune cells within tumor cell populations that are associated with poor prognosis [21,22]. Furthermore, Olbrecht et al. performed scRNAseq on 18,403 cells collected from 7 subjects and identified cell phenotypes originating from the primary tumor and metastatic lesions. Their results suggested that intratumor heterogeneity may be at least partially due to differences in stromal cells and suggested marker genes on these cells as possible therapeutic targets [23]. Our hypothesis is that deconvolution of bulk RNA-seq data will reveal cancer cell populations that are associated with clinical outcomes, such as survival, response to chemotherapy, or optimal surgical cytoreduction. The molecular make-up of cell populations correlated with worse outcomes may be useful for designing alternative targeted therapeutic strategies. To test our hypothesis, we aimed to identify HGSC cell populations by deconvoluting bulk RNA-seq data and assessing their associations with clinical outcomes. Deconvolution was performed using gene expression patterns and a publicly available reference set of scRNAseq data from benign fallopian tubes and HGSC. Then, we identified the molecular characteristics of the cell populations associated with clinical outcomes. Validation of the results was performed in publicly available datasets, including TCGA [2].

## 2. Results

**Figure 1:**
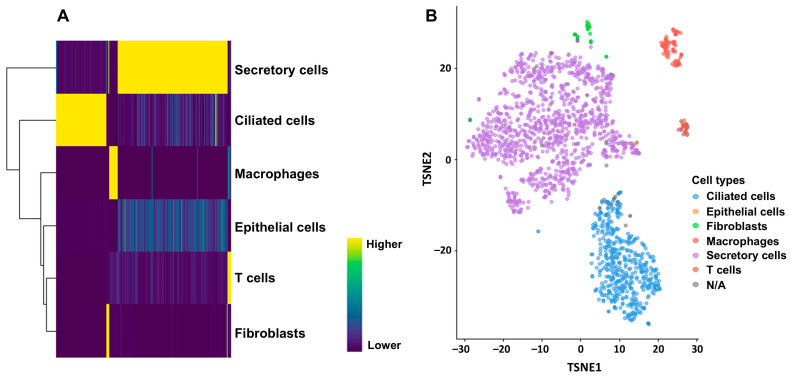
Annotation of cells included in the GSE139079 scRNAseq experiment using the SingleR package (v 2.6.0). (**A**) All cells were annotated against cell types from the GSE189955 scRNAseq dataset. Heatmap based on the matrix of gene log-expression values for different cell types. (**B**) Dimensionality reduction with t-SNE plotted using the plotTSNE function (from the scater R package, v 1.36.0). Each point represents a cell and is colored according to its cell type label based on the HPCA reference. Fallopian tube scRNA-seq revealed a majority of differentiated ciliated and secretory cells (over 90%), a few (7%) immune cells, and 0.6% epithelial cells.

**Figure 2:**
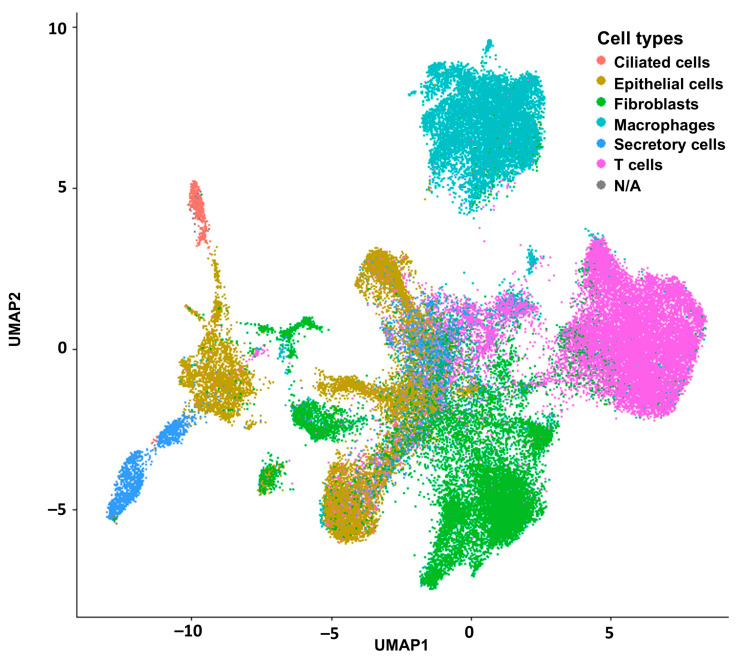
Integration of all reference scRNAseq experiments with the Seurat package (v 5.3.0) for posterior deconvolution.

**Figure 3:**
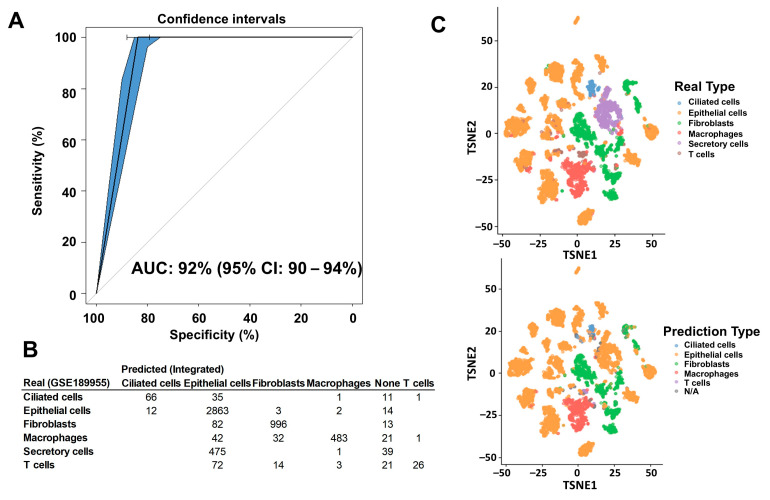
Comparison of cell type annotation between the integrated dataset and the GSE189955 set. The database extracted from GSE189955 was annotated by the authors themselves. The integrated database included data from studies GSE189955, GSE139079, and GSE154600. We annotated all cells of the integrated dataset based on the initial cell annotation from the GSE189955 scRNAseq experiment because they have the same cellular types included in the integrated database: normal fallopian tube cells and HGSC cells. (**A**) We used the integrated set to predict cellular type proportion of the GSE189955 dataset, and we used the actual real cellular classification to measure the prediction performance of the integrated set. The prediction performance, measured by the AUC of the integrated set was 92% (95% CI: 90–94%). (**B**) Table of real GSE189955 annotated cell types, versus predicted cell types annotated with the integrated dataset. (**C**) When using the integrated set for annotation, secretory cells were classified as epithelial cells, with no other specification, as it could be visualized when comparing the upper panel (real classification), with the lower panel (predicted classification) where the purple dots disappear (secretory cells). Other cells seemed to be classified quite accurately.

To carry out the deconvolution of our bulk RNA-seq data, we used diverse databases as references. The annotation of these databases was performed with different methods that affected the results of the deconvolution and the association of cell type proportions with clinical outcomes. First, we annotated the reference database HPCA, which is a general-purpose classification tool for scRNAseq experiments. The GSE139079 scRNAseq study annotations are shown in Supplementary Figure S1, and the GSE189955 data are shown in Supplementary Figure S2. However, given the similarity of the GSE189955 samples with our own data (normal tube cells and HGSC), we decided to use the cell type classification by the authors as an annotation for GSE139079 (Figure 1), GSE154600, and HPCA (Supplementary Figure S3) and for the integrated database encompassing all three datasets: GSE189955, GSE139079 and GSE154600 (Figure 2). To assess how well the new integrated dataset was able to annotate other databases, we used this integrated set to predict the cell types from the GSE189955 dataset. Then, we compared the predicted classification with the real values of GSE189955. The AUC of the predicted classification was AUC of 92% (95% CI: 90–94%, Figure 3A). It seemed that when using the integrated set for annotation, secretory cells were classified as epithelial cells (Figure 3B), as could be visualized when comparing the upper panel of Figure 3C (real classification) with the lower panel (predicted classification). Other cells seemed to be classified accurately.

### 2.1. Deconvolution of Bulk RNAseq Revealed Fibroblasts, Immune Cells and Epithelial Cells in the UI Dataset Which Was Similar in the TCGA Dataset

When we used the HPCA dataset as a reference for deconvolution of the UI dataset, we detected more epithelial cells in both the normal fallopian tube and HGSC samples. HGSC samples presented more immune and connective tissue cells (Supplementary Figure S5A). Using the scRNAseq dataset GSE189955 (with samples similar to our experiment) as a reference for deconvolution, in normal FT (fallopian tube) cells, we detected a predominance of ciliated and secretory cells that diminished in HGSC samples in favor of fibroblasts and other epithelial cells (Supplementary Figure S5B). In the TCGA dataset deconvolution, we observed similar cellular proportions to ours: epithelial cells were predominant when annotated with HPCA (Supplementary Figure S6A), while fibroblasts dominated the cell classification when GSE189955 was used as a reference (Supplementary Figure S6B). The TCGA cohort included only HGSC samples.

**Figure 4:**
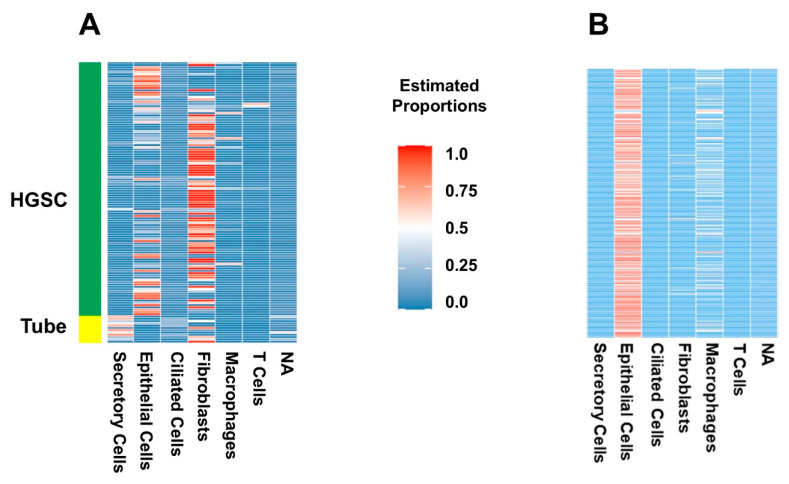
Deconvolution of bulk UI and TCGA RNA-seq experiments with patterns from integrated scRNA-seq experiments as a reference (using MuSiC). (**A**) Deconvolution of UI bulk RNA-seq data using the integrated dataset (GSE189955, GSE139079, and GSE154600) as reference. Heatmap of estimated proportions of cells (vertical) for each sample (horizontal). For the deconvolution analysis of the UI specimens, we included both HGSC and normal FT samples. Secretory cells are present in FT samples from the cohort, while they were almost entirely not present in HGSC samples. The HGSC samples had more epithelial cells (not otherwise typified), immune cells and fibroblasts. (**B**) Deconvolution of TCGA bulk RNAseq of HGSC using the integrated dataset (GSE189955, GSE139079, and GSE154600) as reference. Heatmap of the estimated proportions of patient samples (horizontal) and each cell type (vertical). The TCGA cohort included only HGSC samples. As with the UI dataset, there is an abundance of epithelial cells (not otherwise typified), immune cells and fibroblasts (in that order), with scant numbers of ciliated and secretory cells.

Finally, we used the integrated dataset as a reference for the deconvolution of our experiment (Figure 4). Secretory and ciliated cells were present in normal FT samples from the cohort, while they were almost entirely not present in UI HGSC samples, which the integrated set annotation method tended to do when applied to HGSC cells (Figure 3). The UI HGSC samples had more epithelial cells (not otherwise typified), immune cells and fibroblasts. To assess how well the integrated set classified population cells we used the GSE189955 dataset, because it was the one most similar to our set. Then, we estimated GSE189955 cell proportions, deconvoluting with the integrated set, and compared them with the real cell proportions, resulting in a prediction performance of an AUC of 92% (95% CI: 90–94%, Figure 3A). Deconvolution of TCGA bulk RNAseq of HGSC showed a similar cell distribution as that of the UI dataset, with an abundance of epithelial cells, immune cells and fibroblasts (in that order) and a very small number of ciliated and secretory cells (Figure 4B). Fibroblasts were more abundant in the UI samples. Interestingly, after deconvolution of TCGA bulk RNA-seq data using GSE189955 as a reference (Supplementary Figure S7B), cell proportions were most like cell proportions after deconvolution of UI RNA-seq HGSC samples using either the integrated set as a reference (Figure 4A) and/or the GSE189955 set as a reference (Supplementary Figure S6B).

### 2.2. Certain Cell Types Were Associated with Worse Patient Outcomes Depending on the Dataset and Analysis Used

Clinical variables, including preoperative and surgical features as well as clinical outcomes, are detailed in Table 1. The proportions of cell types with clinical data were included in association studies. First, we determined the association of the cell proportion with the presence of HGSC. According to the multivariate model, a higher amount epithelial cells and a lower amount of secretory cells remained significant after accounting for other cell proportions (Table 2). Since the integrated annotation tended to classify ciliated cells as epithelial cells (Figure 3B, 5th row), there was a higher amount of epithelial cells. A lower number of secretory cells is expected, as this is a normal component of intact fallopian tubes. However, when cell proportions between FT tissue and HGSC tissue were compared with those in the GSE189955 annotation, we still observed a significantly lower amount of ciliated and secretory cells (Supplementary Figure S7B). Comparisons with cell proportions based on the HPCA revealed a higher amount of NK cells and chondrocytes (Supplementary Figure S7A). To our knowledge, and after a review of the literature, a higher amount of chondrocytes in the tumor microenvironment has not been shown to be associated with ovarian cancer, and the significance of these findings in our study is unclear. On the other hand, NK cells are known to be increased in the tumor microenvironment in ovarian cancer and have early cytolytic activity on cancer cells, and several therapies have been tested in clinical trials to recruit NK cells in ovarian cancer using IL-15 and IL-2 with limited success [24].

An increasing proportion of fibroblasts was negatively associated with overall survival (*p* = 0.013, hazard ratio (HR) = 2.6) according to univariate analysis of the GSE189955 dataset (Supplementary Figure S8). A decrease in the number of ciliated cells tended to be positively associated with survival (*p* = 0.053, hazard ratio (HR) < 0.001) according to the univariate analysis based on the integrated set annotation (Supplementary Figure S9). None of these cell proportions were significant in multivariate analyses including the following clinical variables: age at diagnosis (*p* = 0.046) and response to chemotherapy (*p* < 0.001).

When assessing the associations with clinical outcomes in patients with HGSC, age at diagnosis, the Charlson Comorbidity Index, residual disease after surgery, and neoadjuvant chemotherapy were significant (*p* < 0.05) in the univariate analysis of clinical features associated with response to chemotherapy. After adjusting for all these factors, residual disease after surgery (*p* = 0.030) and after neoadjuvant therapy (*p* = 0.010) remained significant. According to the univariate analysis of clinical features associated with optimal surgery (including all variables from Table 1), only disease in the chest trended toward significance (*p* = 0.067). No cellular types, based on the integrated set annotation, were significantly associated with either response to chemotherapy or surgical outcomes (Supplementary Table S1). However, a higher proportion of macrophages was associated with the response to chemotherapy (based on the GSE189955 annotation) in the univariate analysis (Table 3) and in the multivariate analysis of the classification based on the HPCA annotation (Supplementary Table S2). Cell proportions based on the GSE189955 and HPCA datasets were not associated with surgical outcomes.

In the TCGA dataset, when deconvoluted and annotated with the integrated set, epithelial cells were associated with a response to chemotherapy, and fibroblasts were associated with a nonresponse to chemotherapy (Supplementary Table S4). According to the multivariate analysis, increasing proportions of fibroblasts were associated with worse surgical outcomes after adjusting for available clinical features. Supplementary Table S3 shows the clinically available characteristics, and Supplementary Table S4 shows the results of the association analyses with clinical outcomes.

### 2.3. Pathway Enrichment Analysis Showed Immune Pathways Were Activated

After extracting the molecular characteristics of each resulting cellular type, we performed pathway enrichment analyses for the cell types annotated with GSE189955 and with the integrated database. Supplementary Table S5 includes significant gene expression features (*p* < 10^−4^) and characteristics of all cellular types resulting from GSE18995 deconvolution of the UI dataset and used for pathway enrichment analyses. We assessed significant biological processes related to all cell types but specifically to those cellular types associated with clinical outcomes (see the previous section).

**Figure 5:**
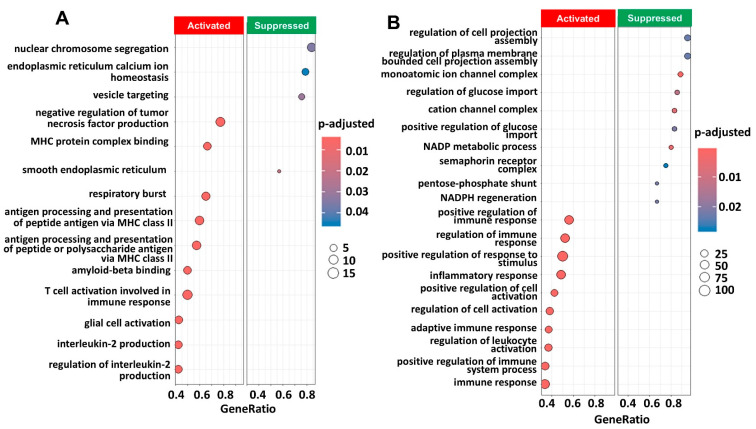
Pathway enrichment analysis of genes obtained by scRNA seq associated with macrophages annotated clusters from scRNAseq from GSE189955 using the GO database with clusterProfiler (v 4.12.6). (**A)** Pathway enrichment analysis using the characteristic genes resulting from the annotation of macrophages (*p* < 10^−4^) derived from the GSE189955 dataset. We show the most significant pathways that are activated (n = 10) and suppressed (n = 4) in the enrichment analysis. The *p* value was adjusted for FDR (p.adjust). (**B**) Same pathway analysis but resulting from characteristic gene expression from the integrated dataset: GSE139079, GSE189955, and GSE154600. We show the most significant pathways that are activated (n = 10) and suppressed (n = 10) in the enrichment analysis. A complete list of significant GO pathways is provided in Supplementary Table S6. Gene ratio: gene abundance within macrophages versus non-macrophages. Count: number of significant genes participating in the GO pathway.

Univariate analysis revealed that macrophages were associated with the response to chemotherapy in the GSE189955 dataset, and multivariate analysis revealed that macrophages were associated with the response to chemotherapy in the HPCA dataset. Figure 5 shows all the significant GO pathways associated with the molecular features of macrophages based on the GSE189955 (Figure 5A) and integrated datasets (Figure 5B). It seems that immune pathways were activated in both instances, like GO:0006954 or ‘inflammatory response’ (*p* = 0.004), GO:0002286 or ‘T-cell activation involved in immune response’ (*p* < 0.001), GO:0002263 or ‘cell activation involved in immune response’ (*p* = 0.004), and GO:0050865 or ‘regulation of cell activation’ (*p* = 0.004). Pathways related to intracellular transportation and the cytoskeleton were suppressed, like GO:0032469 or ‘endoplasmic reticulum calcium ion homeostasis’ (*p* = 0.046), GO:0006903, ‘vesicle targeting’ (*p* = 0.029), and GO:0043408 or ‘regulation of MAPK cascade’ (*p* = 0.02). A full list of FDR-adjusted GO pathways is provided in Supplementary Table S6. Higher levels of epithelial cells and lower levels of ciliated cells were associated with ovarian cancer samples. Cilia function and structure were activated by ciliated cell gene features (GSE189955 annotation, Supplementary Figure S10A) and cellular membrane and signal transduction (HPCA annotation, Supplementary Figure S10B), and mitochondrial assembly and function were suppressed. Cell migration, motility and adhesion were activated based on pathway analysis of epithelial cell gene features (GSE189955 and HPCA annotations, Supplementary Figure S11), and intracellular metabolism was suppressed (HPCA annotation, Supplementary Figure S11B). The GO pathways associated with fibroblast and T-cell gene features are detailed in Supplementary Figures S12 and S13.

## 3. Discussion

Despite having similar disease stages, types of tumors, disease spread, and treatments, some patients will experience disease recurrence, while others may experience prolonged disease-free periods. Ovarian cancer is known to be a heterogeneous disease, and our study aimed to identify HGSC cell populations by deconvoluting bulk RNA-seq data and assess their associations with clinical outcomes.

The initial conclusion is that deconvolution results depend on the reference database used for deconvolution. We tried 3 different annotations: (1) HPCA with a comprehensive panel of cell types [25] but not specific for HGSC; (2) a specific HGSC-annotated HGSC reference, GSE189955 [26], with cells from patients with HGSC; and (3) an integrated database of 3 different scRNAseq datasets, GSE139079, GSE189955, and GSE154600, which were also annotated with GSE18995 cell types. When deconvolution of the UI dataset bulk RNA-seq database was performed with the comprehensive HPCA database, a few cellular types that are not usually found in the tube or in HGSC were found, such as chondrocytes, albeit at small percentages. The significance of chondrocytes is unclear in our findings and has not been shown previously to our knowledge. Other cellular types, such as smooth muscle cells and a variety of immune cells, were also observed after this deconvolution. To confirm these findings, parallel bulk RNA-seq and scRNA-seq experiments are needed.

Deconvolution after annotation with the integrated database had similar cell proportions to GSE189955 when used as a reference, with a prediction performance of cellular classification measured by an area under the curve (AUC) of 92% (95% CI: 90–94%). The misclassification of secretory cells in the initial annotation of the integrated dataset was most likely the cause of the decrease in performance, as seen in the section on scRNAseq annotation. We could conclude, however, that both sets would be adequate for use as a reference for the deconvolution of bulk RNA-seq data from HGSC samples and controls.

When analyzing the associations of cell types with cancer diagnosis, we observed that there were significantly higher levels of epithelial cells and significantly lower levels of secretory cells in a multivariate model associated with HGSC compared to those associated with FT after deconvolution with the integrated dataset and GSE189955 dataset. Additionally, a higher proportion of macrophages was associated with a better response to chemotherapy according to the univariate analysis of the GSE189955 dataset. This association became nonsignificant after adjusting for other covariates. Furthermore, we did not observe an association between macrophages and chemotherapy response in the deconvoluted TCGA dataset. The significance of this should be interpreted with caution, as the TCGA and UI datasets yielded different results. Differences could be explained by how the samples were initially prepared, and by the representative populations of each dataset. The association between tumor-associated macrophages and the response to chemotherapy has not been fully established, with studies supporting better response and prognosis [27] and others showing conflicting results [28].

We observed an association between an elevated proportion of fibroblasts and decreased optimal surgical cytoreduction in the TCGA database. Cancer-associated fibroblasts (CAFs) have been associated with a poor prognosis and resistance to chemotherapy in ovarian cancer patients [29]. We present associations of cellular types with different clinical outcomes. These associations do not indicate causality and must be interpreted with caution. Further functional studies are needed to assess the accuracy of these associations.

Pathway analyses of the expression patterns of cell populations associated with clinical outcomes revealed biological processes and pathways that could be potential targets of that specific cluster or cell type. For example, we observed that macrophages were associated with a better response to chemotherapy. In colon cancer, tumor-associated macrophages (TAMs) are some of the most abundant immune cells present in tumors [28]. The role of TAMs in colon cancer progression is complex, with studies associating them with better prognosis [30], while others predict the response to checkpoint inhibitor therapy [31] but are also associated with poorer prognosis, inducing epithelial–mesenchymal transition, and enhancing invasion, migration and metastasis [28]. Some of the genes involved in the enrichment pathway analysis of the macrophages, including *CD68*, *CD163*, and *STAB1*, participate in significant pathways, such as GO:0006954 or ‘inflammatory response’, and are known TAM markers [28]. Other notable pathways significantly associated with the immune response and associated with macrophages gene features were GO:0002286 (or ‘T-cell activation involved in immune response’), GO:0002263 (or ‘cell activation involved in immune response’), and GO:0050865 (or ‘regulation of cell activation’). These pathways are potential targets for novel immunotherapy with antibodies against PD-1 and PD-L1 checkpoints proteins [32,33]. Additionally, some of the GO pathways found to be significantly associated with the molecular features of macrophages are also potential treatment targets. For example, GO:0043408 (‘regulation of MAPK cascade’) and GO:0070371 (‘ERK1 and ERK2 cascade’) may be targeted pharmacologically in HGSC [34]. Similarly, there have been reports that the MAPK cascade and ERK1/2 pathways interact with the JAK/STAT pathway [35,36,37,38]. The JAK1/STAT3 pathway has been studied extensively in ovarian cancer in multiple models, identifying this pathway as a potential target [39]. Multiple modulators of the JAK/STAT pathway are in development, and some have been studied in phase I/II trials, such as ruxolitinib, which has shown promising in vitro findings [40]. Our data further support the significance of this pathway in ovarian cancer. All these data seems to agree with our initial hypothesis that bulk RNA-seq deconvolution may identify cancer cell populations associated with clinical outcomes and may prove useful for designing alternative targeted therapeutic strategies. However, we are still removed from using this information in the clinical setting. It seems that we can detect cellular types within a bulk tumor sample, but we do not know whether cell types of lower frequency and/or in different states of dormancy may play an important role in resistant disease.

In our study, we identified similar but not identical proportions of cells in the TCGA sample set. Epithelial cells were the most abundant cellular type in the TCGA cohort deconvoluted with the integrated set as a reference, while fibroblasts were more abundant in the UI cohort. After deconvolution of the TCGA dataset with GSE18995 as a reference, cell proportions were the most similar to cell proportions from the deconvoluted UI set with GSE18995 and/or the integrated sets as references. This emphasizes the necessity to study each sample set independently to account for other factors, such as location (primary vs. metastatic site), batch effect (which was accounted for in our analysis), background of the tissue where biopsies were obtained, amount of biopsy taken (more or less inflammatory or support tissue), histology type and grade of tumor, vascular infiltration, etc. In our sample set, only 13% of the samples originated from a location other than the primary tumor (pelvic mass), and the location of the biopsy did not influence clinical outcomes. Additionally, our patient population included a homogenous cohort (Iowa). This could account for the difference in cell populations being more abundant in fibroblasts than in the TCGA cohort. This information may be important to record and analyze in the future, especially when assessing recurrence and resistance to chemotherapy.

To our knowledge, there have been few attempts to use scRNAseq data to deconvolute bulk RNAseq data using multiple existing reference datasets in ovarian cancer. One such study explored deconvolution of bulk RNAseq data from ovarian tumor tissue in a much smaller sample size (n = 8) [41]. Our study offers a much larger HGSC sample size as well as normal FT samples.

One of the strengths of our study is that the study was carried out at a single institution with a similar treatment philosophy and the same methods for collecting and processing specimens. This resulted in a homogeneous database of HGSC samples. Additionally, all genomic data are clinically annotated, and samples correspond to patients who have undergone long-term surveillance and clinical outcome assessment. Bulk RNAseq deconvolution was performed with different reference annotations, GSE189955, HPCA, and an integrated database containing more than 59,000 cells. The diversity of cellular types and annotations gave us a variety of potential classifications for our bulk experiment. We ended up referencing the datasets annotated with cell types that were common to our own experimental design (GSE18995 and integrated database) that were composed of normal FT samples and HGSC samples. Moreover, our impression is that the best possible outcomes would be accomplished with an experiment that combines scRNA-seq and bulk RNA-seq to properly classify all cellular types (scRNA-seq) while still obtaining some of the benefits of global gene expression with additional knowledge on alternative splicing, point mutations, novel transcripts, long noncoding RNAs and gene fusions via a simple and cost-effective technique (bulk RNA-seq). Newer emerging technologies [42], such as spatial RNA sequencing (spRNAseq) and analysis of digitalized slides from tumor samples using artificial intelligence (AI), may revolutionize this field, identifying cells with the worst prognosis in tumors.

Limitations of our study include its retrospective design. Our original bulk RNA-seq experiments were not designed to assess tumor cell populations, and the data can suffer from a batch effects related to sample processing. The study is also purely in silico, and there is a risk of false positives due to multiple testing. Thus, despite the statistical significance of the association between different cell types and cancer and the association with clinical outcomes, these results will have to be examined and validated in a prospective manner when the design of the experiments is specific for this objective, namely, with scRNAseq experiments. Additionally, samples from UI were extracted from the population that it serves, and Iowa has a predominant white composition, mainly with Northern European ancestry (90.1%) [43]. Some of the analyses are based on limited controls (samples from normal FT), which could serve as a source of bias.

In summary, in our deconvolution study of bulk RNA-seq HGSC samples, we identified cell populations within the tumor that may be associated with some of the observed clinical outcomes. If these results were confirmed in a prospective study with a study design specific for the objectives, we could have new possibilities for a more personalized and specific treatment, either surgical or cytotoxic, for each patient.

## 4. Methods

We performed a retrospective, single-institution study in which we included stage III and IV patients with HGSC. RNA was extracted from tumor specimens and processed as detailed below to obtain the necessary genomic data. The study protocol was approved by the University of Iowa Institutional Review Board (IRB), including human subjects/materials, on 25 April 2018 (IRB201804817: ‘Prediction Models in Ovarian Cancer’). The University of Iowa Department of Obstetrics and Gynecology maintains the Women’s Health Tissue Repository (WHTR), which contains more than 60,000 biological samples, including more than 2500 primary gynecologic tumors. All tissues in the WHTR were collected from 1990 to 2014 after informed consent was obtained from the patients, and the study was carried out in accordance with University of Iowa IRB guidelines (IRB Number 200910784 and IRB Number 200209010). Genomic data from HGSC patients at our institution were compared with publicly available RNAseq data from HGSC patients.

### 4.1. Data and Specimen Procurement

Benign fallopian tube (FT) cell scRNAseq data were extracted from the bioproject GSE139079 (n = 1765) [44], HGSC scRNAseq data were extracted from the bioproject GSE189955 (n = 5329) [26], and HGSC scRNAseq data were extracted from the bioproject GSE154600 (n = 52,121). We chose these databases because they were extracted from the same type of tissue as our bulk RNAseq experiment. All were used as references for further analyses. Additional data were obtained from the TCGA high-grade serous cancer dataset (n = 373, dbGaP #29868) [2].

HGSC tissue samples (n = 112) and FT data (n = 12) and clinical data were collected from the Department of Obstetrics and Gynecology Gynecologic Oncology Biobank (termed Biobank) (IRB, ID#200209010), which is part of the Women’s Health Tissue Repository (WHTR, IRB, ID#201804817) and available in the repository GSE156699. Acquisition was described in a previous study [5]. HGSCs were collected from patients with stage III and IV HGSC, and FTs were collected from healthy individuals without any personal or family history of cancer. All tissues archived in the Gynecologic Oncology Biobank were originally obtained from adult patients who provided informed consent in accordance with University of Iowa (UI) IRB guidelines. Tumor samples were collected, reviewed by a board-certified pathologist, and flash-frozen, and the diagnosis was confirmed in paraffin at the time of initial surgery. Clinical data, including outcomes (survival, response to chemotherapy, surgical outcomes), from HGSC patients were extracted from the electronic medical records in accordance with IRB guidelines (Table 1). The responders to chemotherapy were HGSC patients with a progression-free survival of at least 6 months after the first platinum-based treatment. Nonresponders were those who did not respond (platinum-resistant) or who progressed during treatment (platinum-refractory). Surgical outcomes were assessed based on the amount of residual disease at the time of surgery. We classified surgical outcomes as no residual disease after surgery (R0), no residual lesions greater than 1 cm in size (R1 or also known as ‘optimal cytoreduction’), and suboptimal debulking, residual disease of more than 1 cm, or R2.

RNA was then isolated from these tumor specimens. RNA extraction, processing and sequencing were performed as described previously [5,45]. In brief, total cellular RNA was extracted from primary tumor tissue using the mirVana (Thermo Fisher, Waltham, MA, USA) RNA purification kit. The RNA yield and quality were assessed with a Trinean Dropsense 16 spectrophotometer and an Agilent Model 2100 bioanalyzer. The RNA quality was determined to be adequate if the sample had an RNA integrity number (RIN) of 7.0 or greater. Samples that were of adequate quality were then sequenced. Five hundred nanograms of RNA were quantified with a Qubit spectrophotometer (Thermo Fisher). RNA was then converted to cDNA and ligated to sequencing adaptors with Illumina TriSeq stranded total RNA library preparation (Illumina, San Diego, CA, USA). cDNA samples were then sequenced with the Illumina HiSeq 4000 genome sequencing platform using 150 bp paired-end SBS chemistry. All sequencing was performed at the Genome Facility at the University of Iowa Institute of Human Genetics (UIHG).

### 4.2. Data Preprocessing

Subread (v 2.0.6) was used to align the RNA-seq reads to the human reference genome (version hg38) [46]. We created BAM files after alignment. FeatureCounts (v 2.0.6) was used to measure gene expression [47]. The DESeq2 package (v 1.44.0) was used to import, normalize, and prepare the gene counts for analysis [48]. Gene expression was independently used for the association analysis. ENSEMBL was used to annotate gene expression alignment analysis. RNA-seq data from the bioproject GSE139079, GSE189955, GSE154600, and TCGA HGSC datasets were also preprocessed in a similar way to determine gene expression.

### 4.3. Data Analysis

Annotation of scRNA-seq cell populations: We first identified populations of HGSC cells in RNA-seq experiments from frozen surgical specimens based on gene expression patterns and reference sets from scRNA-seq. After the datasets were downloaded, normalized and log2 transformed, gene expression was determined using DESeq2 (v 1.44.0) software [48]. Briefly, the scRNAseq data were aligned to the hg38 genome with Subread (v 2.0.6). Cells from the GSE189955 scRNA-seq experiment have already been annotated. To annotate cells included in the GSE139079 scRNAseq experiment, we used SingleR (v 2.6.0), an automatic annotation method that, given a reference dataset of samples with known labels, classifies new cells from a test dataset based on gene expression similarity to the reference [49]. The tool will train and test the cell classification based on cluster profiles of gene expression. For this specific instance, we used both (1) the Human Primary Cell Atlas (HPCA) a general pourpose cell classification tool and the built-in reference set as implemented by the package celldex (v 1.14.0) (Supplementary Figure S1) [25] and (2) cell types from the GSE189955 scRNAseq experiment with HGSC cells that were already annotated (Figure 1). We also annotated cells from the GSE154600 scRNAseq experiment with SingleR (v 2.6.0) using the HPCA (Supplementary Figure S2) and GSE189955 as a reference (Supplementary Figure S3).

We used the Seurat package (v 5.3.0) [50] to integrate all three downloaded datasets: GSE139079 (with 1765 cells), which included benign fallopian tubes; GSE189955 (with 5329 cells), which included mainly HGSC cells; and GSE154600 (with 52,121 cells), which included HGSC cells [15]. Seurat’s integration methods identify and correct batch effects or other sources of unwanted variation across different scRNA-seq datasets. The integration procedure implements a dimensional reduction that captures the shared sources of variance across multiple layers so that cells in a similar biological state will cluster. Finally, we annotated this integrated scRNA-seq database using cell types from the GSE189955 scRNA-seq experiment (Figure 2, Supplementary Figure S4). Based on the study descriptions of the three included databases, we concluded that this approach was the best option for this integrated set.

To assess the performance of the classification predictions, we used the pROC R package (v 1.18.5) for visualization of the receiver operating characteristic (ROC) curves, area under the curve (AUC), and confidence intervals [51].

### 4.4. Deconvolution of Bulk RNA-seq from HGSC Datasets

Deconvolution is a process intended to determine the composition of each sample analyzed based on a reference cluster classification. Deconvolution was performed with the MUlti-Subject SIngle Cell Deconvolution (MuSiC, v1.0.0) method, which utilizes cross-subject scRNA-seq to estimate cell type proportions in bulk RNA-seq data. We used this method because it has outperformed existing methods, especially for tissues with closely related cell types [16]. To deconvolute our bulk RNA-seq data, we used several strategies. First, we used two different sets of scRNAseq data as references: (1) a scRNAseq dataset of normal fallopian cells (GSE139079) and (2) a scRNAseq dataset of HGSC cells from an independent study (GSE189955). Then, we used an integrated database created from three downloaded databases: GSE139079, GSE189955, and GSE154600. Gene expression and cell annotation of the referenced databases were used to estimate cell type proportions from bulk sequencing data from patients with HGSC. Deconvolution and estimation of cell type proportions were performed in datasets from the UI and TCGA HGSC repositories. Before deconvolution of the TCGA datasets, we adjusted gene expression for batch effects using the ComBat_seq function for count data (sva R package, v 3.52.0) [52].

### 4.5. Comparison of Patient Outcomes to Clustered Cell Proportions

After deconvoluting each sample of the datasets in the study, we obtained a percentage of cellular types for each HGSC specimen based on the reference database cells. This information was added to the clinical variables, including epidemiologic data, patient outcomes, survival, response to chemotherapy and whether optimal cytoreduction was achieved (see Table 1). We then compared clustered-cell percentage and other clinical variables to assess whether there were differences in clinical outcomes and treatment results. Univariate and multivariate logistic regression analyses were performed to assess these associations. Only variables with a *p* value around or less than 0.10 in univariate analyses were included in multivariate models. Stepwise reduction in the number of variables was used to create the best fitting multivariate final model (using the step R function). We used R^2^ to assess how well our final model fits the data. Univariate and multivariate Cox proportional hazard ratios were calculated to assess the associations of survival with clustered-cell proportions and clinical variables. As with the logistic regression analysis, variables with a *p*-value around or less than 0.10 in Cox univariate analyses were included in multivariate proportional hazard models. Stepwise reduction in the number of variables was used to create the best fitting multivariate model (using the step R function). Significant associations were considered those with a *p* value < 0.05.

The database extracted from the bioproject GSE189955 was annotated by the authors themselves. We integrated datasets extracted from the GSE189955, GSE139079, and GSE154600 datasets. The integrated database included more than 59,000 ovarian cancer and fallopian tube cells with more than 74,000 gene expression features. We annotated all cells of the integrated dataset based on the initial cell annotation from the GSE189955 scRNAseq experiment.

Uniform manifold approximation and projection (UMAP), a dimension reduction technique, was used for visualization of this dimensional reduction. Each point represents a cell and is colored according to its cell type label based on the GSE189955 cell-specific reference. In Supplementary Figure S4, we present the same visualization of this dimensional reduction annotated by databases (GSE189955, GSE139079, and GSE154600).

### 4.6. Pathway Enrichment Analysis

To further characterize the molecular characteristics of each resulting cellular type, we performed a pathway enrichment analysis using the most informative genes for each cellular type using the FindMarkers feature from the Seurat package (v 5.3.0): only genes that were differentially expressed for each of the identity classes (cellular types) with a *p*-value < 10^−4^ to account for multiple comparisons were included in pathway enrichment analyses that query the GO database with clusterProfiler (v 4.12.6) [53]. All analyses were performed using the R environment for statistical computing and graphics (www.r-project.org, version 4.3.1) [54]. Significant pathways were considered after adjusting for the false discovery rate (FDR).

## Figures and Tables

**Table 1:** Patient characteristics of the UI dataset.

		HGSC (N = 112)	Range
Preoperative characteristics	Age (mean)	60	25–85
	BMI (mean)	27.2	14.4–54.2
	Charlson Comorbidity Index *		
	Low (1–3)	8	
	Medium (4–6)	63	
	High (>6) Not available	8 33	
	Preop CA-125 (mean)	2414	7–32,865
	Disease in Upper abdomen (Other than Omentum) by Imaging	67	
	Large bowel	4	
	Spleen	0	
	Mesenteric LN	4	
	Porta/Hepatis	5	
	Other	28	
	Disease in the Chest by Imaging		
	Tumor	7	
	Pleural effusion	5	
	Neoadjuvant chemotherapy		
	Yes	14	
	No	91	
Operative characteristics	FIGO Stage		
	I–II	4	
	III	71	
	IV Not available	28 9	
	Omentectomy	102	
	Surgery large bowel	32	
	Splenectomy	2	
	Diaphragmatic stripping	6	
	Residual disease		
	Microscopic (R0)	21	
	<1 cm (R1)	38	
	Macroscopic (R2) Not available	36 17	
	Surgical complexity score ^#^		
	Low	55	
	Intermediate	51	
	High Not available	5 1	
Outcomes	30-day mortality	0 (0%)	
	90-day mortality	1 (1%)	
	Response to chemotherapy **		
	Yes	50	
	No Not available	39 23	

* The Charlson Comorbidity Index is a measure of the prognostic burden of all associated morbidities to predict mortality and is the most validated measure of the prognostic impact of multiple chronic illnesses (www.charlsoncomorbidity.com). ^#^ Surgical complexity score: score to predict surgical morbidity and 90-day mortality after primary debulking surgery for HGSC. ** Response to chemotherapy: Responders: no recurrence for at least 6 months after finishing treatment. Non-responders: progressed during treatment (refractory to therapy), whose tumors were not modified significantly by treatment, or who progressed within 6 months after finishing treatment (resistant to therapy).

**Table 2:** Association analysis of cell proportions with ovarian cancer in the UI dataset after deconvolution.

	Cell Proportions		Univariate		Multivariate	
	HGSC	Tube	OR	*p* Value	OR	*p* Value
Ciliated cells	0.03	0.13	2.6 × 10^−15^	<0.001 *	**1.88**	0.969
Epithelial cells	0.30	0.01	4.1 × 10^9^	0.033 *	2.3 × 10^8^	0.042 *
Fibroblasts	0.49	0.24	9.40	0.029 *	1.68	0.955
Macrophages	0.04	0.003	4.3 × 10^7^	0.366	NS	NS
N/A	0.05	0.14	3 × 10^−23^	<0.001 *	2.9 × 10^−10^	0.342
Secretory cells	0.09	0.48	0.006	<0.001 *	8.5 × 10^−6^	<0.001 *
T cells	0.01	0.00	1.1 × 10^81^	0.993	NS	NS

When comparing the proportion of cell types between HGSC and normal FT after deconvolution with the integrated dataset as a reference, in the univariate analysis, there was a significant decrease in secretory and ciliated cells in HGSC (*p* < 0.05), with a significantly higher amount of fibroblasts and epithelial cells (not otherwise typified). The number of unclassified cells decreased. According to the multivariate analysis, there was a lower amount of secretory cells, and a significantly increased number of epithelial cells after accounting for the other cell types. The R^2^ of this model was 0.71. * Statistically significant (*p* value < 0.05). NS: nonsignificant in the univariate analysis and not introduced in the multivariate model. OR: odds ratio. N/A: Unclassified cells.

**Table 3:** Association analysis of cell proportions with response to chemotherapy and optimal surgical debulking in the UI dataset.

Response to Chemotherapy			Univariate		Multivariate	
	Responders	Non-Responders	OR	*p* Value	OR	*p* Value
Ciliated cells	0.15	0.13	0.42	0.494	NS	
Epithelial cells	0.24	0.22	0.58	0.658	NS	
Fibroblasts	0.43	0.49	2.35	0.271	NS	
Macrophages	0.08	0.04	2.2 × 10^−4^	0.039 *	2.2 × 10^−4^	0.067
Secretory cells	0.03	0.06	12.17	0.263	NS	
T cells	0.07	0.06	0.42	0.778	NS	
**Clinical features**						
Age			1.05	0.011 *	1.04	0.055
BMI			1.05	0.160	NS	
Charlson Comorbidity Index			0.11	0.068	0.461	0.564
Preop CA125			1.00	0.874	NS	
Disease upper abdomen			2.28	0.076	1.194	0.232
Disease in chest			2.7 × 10^−8^	0.991	NS	
Neoadjuvant chemotherapy			8.39	0.009 *	14.21	0.007 *
FIGO Stage			2.67	0.069	1.849	0.065
Optimal surgery (R0 + R1)			0.46	0.097	0.406	0.172
Residual micro (R0)			0.26	0.048 *	0.15	0.027 *
Surgical Complexity score			2.7 × 10^−8^	0.990	NS	
		**Optimal surgery**	**Univariate**		**Multivariate**	
	**Optimal**	**Suboptimal**	**OR**	***p* value**	**OR**	***p* value**
Ciliated cells	0.13	0.16	2.67	0.406	NS	
Epithelial cells	0.22	0.24	2.01	0.537	NS	
Fibroblasts	0.45	0.43	0.81	0.762	NS	
Macrophages	0.09	0.05	0.01	0.108	0.09	0.279
Secretory cells	0.05	0.05	0.73	0.873	NS	
T cells	0.06	0.07	2.20	0.763	NS	
**Clinical features**						
Age			0.99	0.587	NS	
BMI			1.01	0.638	NS	
Charlson Comorbidity Index			1.06	0.946	NS	
Preop CA125			1.00	0.572	NS	
Disease upper abdomen			1.27	0.564	NS	
Disease in chest			4.79	0.069	4.90	0.066
Neoadjuvant chemotherapy			8.39	0.558	NS	
FIGO Stage			7.7 × 10^6^	0.989	NS	
Optimal surgery (R0 + R1)			8.4 × 10^−24^	0.999	NS	
Residual micro (R0)			1 × 10^−8^	0.990	NS	
Surgical Complexity score			0.53	0.508	NS	

Only macrophages were associated with a positive response to chemotherapy after GSE189955 deconvolution in the univariate analysis (*p* = 0.039). According to the univariate analysis, the clinical features associated with response to chemotherapy, or risk of disease relapse, were age at diagnosis, residual disease after surgery, and receiving neoadjuvant chemotherapy (*p* < 0.05). After adjusting for all these factors, residual disease after surgery (*p* = 0.027) and receiving neoadjuvant therapy (*p* = 0.007) remained significant. There was no significant difference in the number of macrophages in this multivariate model, but there was a close trend (*p* = 0.067). No cellular type or clinical features were associated with cytoreduction after GSE189955 deconvolution. We built a multivariate model with those variables with a *p* value around or lower than 0.1 in the univariate model. No variable was significant in the multivariate model. * Statistically significant (*p* value <0.05). OR: odds ratio.

## Data Availability

The datasets generated and/or analyzed during the current study are available in the GEO at NCBI repository: https://www.ncbi.nlm.nih.gov/geo/. Accession numbers: GSE139079, GSE189955, GSE154600 and GSE156699 (for UI HGSC dataset). The validation portion of this study was performed in silico, with deidentified publicly available data. All data from TCGA is available at their website: https://portal.gdc.cancer.gov/ (dbGaP #29868). Software utilized by this study is also publicly available at Bioconductor website: http://bioconductor.org/.

## Supplementary Materials

The following supporting information can be downloaded at https://www.mdpi.com/article/10.3390/ijms26157512/s1.

## Abbreviations

The following abbreviations are used in this manuscript:

AI	artificial intelligence
AUC	area under the curve
Biobank	Department of Obstetrics and Gynecology Gynecologic Oncology Biobank
EOC	epithelial ovarian
FT	fallopian tube
HGSC	High-grade serous cancer
HPCA	Human Primary Cell Atlas
IRB	Institutional review board
MuSiC	MUlti-Subject SIngle Cell deconvolution
RNA-seq	RNA sequencing
ROC	receiver operating characteristic
scRNA-seq	single-cell RNA-seq
spRNAseq	spatial RNA sequencing
TAM	tumor-associated macrophages
TCGA	The Cancer Genome Atlas
UI	University of Iowa
UIHG	University of Iowa Institute of Human Genetics
UMAP	Uniform manifold approximation and projection
WHTR	Women’s Health Tissue Repository

## References

[1] Schwarz R.F., Ng C.K.Y., Cooke S.L., Newman S., Temple J., Piskorz A.M., Gale D., Sayal K., Murtaza M., Baldwin P.J. (2015). Spatial and Temporal Heterogeneity in High-Grade Serous Ovarian Cancer: A Phylogenetic Analysis. PLoS Med..

[2] Bell D., Berchuck A., Birrer M., Chien J., Cramer D.W., Dao F., Dhir R., Disaia P., Gabra H., Glenn P. (2011). Integrated Genomic Analyses of Ovarian Carcinoma. Nature.

[3] Moore K., Colombo N., Scambia G., Kim B.-G., Oaknin A., Friedlander M., Lisyanskaya A., Floquet A., Leary A., Sonke G.S. (2018). Maintenance Olaparib in Patients with Newly Diagnosed Advanced Ovarian Cancer. N. Engl. J. Med..

[4] Ray-Coquard I., Pautier P., Pignata S., Pérol D., González-Martín A., Berger R., Fujiwara K., Vergote I., Colombo N., Mäenpää J. (2019). Olaparib plus Bevacizumab as First-Line Maintenance in Ovarian Cancer. N. Engl. J. Med..

[5] Gonzalez Bosquet J., Devor E.J., Newtson A.M., Smith B.J., Bender D.P., Goodheart M.J., McDonald M.E., Braun T.A., Thiel K.W., Leslie K.K. (2021). Creation and Validation of Models to Predict Response to Primary Treatment in Serous Ovarian Cancer. Sci. Rep..

[6] Gogineni V., Morand S., Staats H., Royfman R., Devanaboyina M., Einloth K., Dever D., Stanbery L., Aaron P., Manning L. (2021). Current Ovarian Cancer Maintenance Strategies and Promising New Developments. J. Cancer.

[7] Gonzalez Bosquet J., Newtson A.M., Chung R.K., Thiel K.W., Ginader T., Goodheart M.J., Leslie K.K., Smith B.J. (2016). Prediction of Chemo-Response in Serous Ovarian Cancer. Mol. Cancer.

[8] Bi J., Thiel K.W., Litman J.M., Zhang Y., Devor E.J., Newtson A.M., Schnieders M.J., Bosquet J.G., Leslie K.K. (2020). Characterization of a TP53 Somatic Variant of Unknown Function From an Ovarian Cancer Patient Using Organoid Culture and Computational Modeling. Clin. Obstet. Gynecol..

[9] Newtson A.M., Devor E.J., Bosquet J.G. (2020). Prediction of Epithelial Ovarian Cancer Outcomes With Integration of Genomic Data. Clin. Obstet. Gynecol..

[10] Bosquet J.G., Marchion D.C., Chon H., Lancaster J.M., Chanock S. (2014). Analysis of Chemotherapeutic Response in Ovarian Cancers Using Publicly Available High-Throughput Data. Cancer Res..

[11] Tothill R.W., Tinker A.V., George J., Brown R., Fox S.B., Lade S., Johnson D.S., Trivett M.K., Etemadmoghadam D., Locandro B. (2008). Novel Molecular Subtypes of Serous and Endometrioid Ovarian Cancer Linked to Clinical Outcome. Clin. Cancer Res..

[12] Verhaak R.G.W., Tamayo P., Yang J.Y., Hubbard D., Zhang H., Creighton C.J., Fereday S., Lawrence M., Carter S.L., Mermel C.H. (2013). Prognostically Relevant Gene Signatures of High-Grade Serous Ovarian Carcinoma. J. Clin. Investig..

[13] Konecny G.E., Wang C., Hamidi H., Winterhoff B., Kalli K.R., Dering J., Ginther C., Chen H.W., Dowdy S., Cliby W. (2014). Prognostic and Therapeutic Relevance of Molecular Subtypes in High-Grade Serous Ovarian Cancer. JNCI J. Natl. Cancer Inst..

[14] Chen G.M., Kannan L., Geistlinger L., Kofia V., Safikhani Z., Gendoo D.M.A., Parmigiani G., Birrer M., Haibe-Kains B., Waldron L. (2018). Consensus on Molecular Subtypes of High-Grade Serous Ovarian Carcinoma. Clin. Cancer Res..

[15] Stuart T., Butler A., Hoffman P., Hafemeister C., Papalexi E., Mauck W.M., Hao Y., Stoeckius M., Smibert P., Satija R. (2019). Comprehensive Integration of Single-Cell Data. Cell.

[16] Wang X., Park J., Susztak K., Zhang N.R., Li M. (2019). Bulk Tissue Cell Type Deconvolution with Multi-Subject Single-Cell Expression Reference. Nat. Commun..

[17] Avila Cobos F., Alquicira-Hernandez J., Powell J.E., Mestdagh P., De Preter K. (2020). Benchmarking of Cell Type Deconvolution Pipelines for Transcriptomics Data. Nat. Commun..

[18] Perets R., Wyant G.A., Muto K.W., Bijron J.G., Poole B.B., Chin K.T., Chen J.Y.H., Ohman A.W., Stepule C.D., Kwak S. (2013). Transformation of the Fallopian Tube Secretory Epithelium Leads to High-Grade Serous Ovarian Cancer in Brca;Tp53;Pten Models. Cancer Cell.

[19] Labidi-Galy S.I., Papp E., Hallberg D., Niknafs N., Adleff V., Noe M., Bhattacharya R., Novak M., Jones S., Phallen J. (2017). High Grade Serous Ovarian Carcinomas Originate in the Fallopian Tube. Nat. Commun..

[20] Ducie J., Dao F., Considine M., Olvera N., Shaw P.A., Kurman R.J., Shih I.-M., Soslow R.A., Cope L., Levine D.A. (2017). Molecular Analysis of High-Grade Serous Ovarian Carcinoma with and without Associated Serous Tubal Intra-Epithelial Carcinoma. Nat. Commun..

[21] Liang L., Yu J., Li J., Li N., Liu J., Xiu L., Zeng J., Wang T., Wu L. (2021). Integration of ScRNA-Seq and Bulk RNA-Seq to Analyse the Heterogeneity of Ovarian Cancer Immune Cells and Establish a Molecular Risk Model. Front. Oncol..

[22] Finotello F., Mayer C., Plattner C., Laschober G., Rieder D., Hackl H., Krogsdam A., Loncova Z., Posch W., Wilflingseder D. (2019). Molecular and Pharmacological Modulators of the Tumor Immune Contexture Revealed by Deconvolution of RNA-Seq Data. Genome Med..

[23] Olbrecht S., Busschaert P., Qian J., Vanderstichele A., Loverix L., Van Gorp T., Van Nieuwenhuysen E., Han S., Van den Broeck A., Coosemans A. (2021). High-Grade Serous Tubo-Ovarian Cancer Refined with Single-Cell RNA Sequencing: Specific Cell Subtypes Influence Survival and Determine Molecular Subtype Classification. Genome Med..

[24] Greppi M., Tabellini G., Patrizi O., Candiani S., Decensi A., Parolini S., Sivori S., Pesce S., Paleari L., Marcenaro E. (2019). Strengthening the Antitumor NK Cell Function for the Treatment of Ovarian Cancer. Int. J. Mol. Sci..

[25] Mabbott N.A., Baillie J.K., Brown H., Freeman T.C., Hume D.A. (2013). An Expression Atlas of Human Primary Cells: Inference of Gene Function from Coexpression Networks. BMC Genom..

[26] Wang Y., Xie H., Chang X., Hu W., Li M., Li Y., Liu H., Cheng H., Wang S., Zhou L. (2022). Single-Cell Dissection of the Multiomic Landscape of High-Grade Serous Ovarian Cancer. Cancer Res..

[27] Zhao B., Pei L. (2023). A Macrophage Related Signature for Predicting Prognosis and Drug Sensitivity in Ovarian Cancer Based on Integrative Machine Learning. BMC Med. Genom..

[28] Song J., Xiao T., Li M., Jia Q. (2023). Tumor-Associated Macrophages: Potential Therapeutic Targets and Diagnostic Markers in Cancer. Pathol. Res. Pract..

[29] Hansen J.M., Coleman R.L., Sood A.K. (2016). Targeting the Tumour Microenvironment in Ovarian Cancer. Eur. J. Cancer.

[30] Pagès F., Mlecnik B., Marliot F., Bindea G., Ou F.-S., Bifulco C., Lugli A., Zlobec I., Rau T.T., Berger M.D. (2018). International Validation of the Consensus Immunoscore for the Classification of Colon Cancer: A Prognostic and Accuracy Study. Lancet.

[31] Mezheyeuski A., Backman M., Mattsson J., Martín-Bernabé A., Larsson C., Hrynchyk I., Hammarström K., Ström S., Ekström J., Mauchanski S. (2023). An Immune Score Reflecting Pro- and Anti-Tumoural Balance of Tumour Microenvironment Has Major Prognostic Impact and Predicts Immunotherapy Response in Solid Cancers. EBioMedicine.

[32] Mirza M.R., Chase D.M., Slomovitz B.M., Christensen R.D., Novák Z., Black D., Gilbert L., Sharma S., Valabrega G., Landrum L.M. (2023). Dostarlimab for Primary Advanced or Recurrent Endometrial Cancer. N. Engl. J. Med..

[33] Eskander R.N., Sill M.W., Beffa L., Moore R.G., Hope J.M., Musa F.B., Mannel R., Shahin M.S., Cantuaria G.H., Girda E. (2023). Pembrolizumab plus Chemotherapy in Advanced Endometrial Cancer. N. Engl. J. Med..

[34] Tyagi K., Roy A., Mandal S. (2023). Pharmacological Inhibition of Protein Kinase D Suppresses Epithelial Ovarian Cancer via MAPK/ERK1/2/Runx2 Signalling Axis. Cell. Signal..

[35] Jin S.H., Choi D., Chun Y.J., Noh M. (2014). Keratinocyte-Derived IL-24 Plays a Role in the Positive Feedback Regulation of Epidermal Inflammation in Response to Environmental and Endogenous Toxic Stressors. Toxicol. Appl. Pharmacol..

[36] Wang T., Liu J., Xiao X.Q. (2015). Cantharidin Inhibits Angiogenesis by Suppressing VEGF-Induced JAK1/STAT3, ERK and AKT Signaling Pathways. Arch. Pharm. Res..

[37] Hong Q., Yang Y., Wang Z., Xu L., Yan Z. (2020). Longxuetongluo Capsule Alleviates Lipopolysaccharide-Induced Neuroinflammation by Regulating Multiple Signaling Pathways in BV2 Microglia Cells. J. Chin. Med. Assoc..

[38] Wang C., Sun H., Zhong Y. (2019). Notoginsenoside R1 Promotes MC3T3-E1 Differentiation by up-Regulating MiR-23a via MAPK and JAK1/STAT3 Pathways. Artif. Cells Nanomed. Biotechnol..

[39] Wen W., Liang W., Wu J., Kowolik C.M., Buettner R., Scuto A., Hsieh M.-Y., Hong H., Brown C.E., Forman S.J. (2014). Targeting JAK1/STAT3 Signaling Suppresses Tumor Progression and Metastasis in a Peritoneal Model of Human Ovarian Cancer. Mol. Cancer Ther..

[40] Han E.S., Wen W., Dellinger T.H., Wu J., Lu S.A., Jove R., Yim J.H. (2018). Ruxolitinib Synergistically Enhances the Anti-Tumor Activity of Paclitaxel in Human Ovarian Cancer. Oncotarget.

[41] Hippen A.A., Omran D.K., Weber L.M., Jung E., Drapkin R., Doherty J.A., Hicks S.C., Greene C.S. (2023). Performance of Computational Algorithms to Deconvolve Heterogeneous Bulk Ovarian Tumor Tissue Depends on Experimental Factors. Genome Biol..

[42] Li X., Wang C.Y. (2021). From Bulk, Single-Cell to Spatial RNA Sequencing. Int. J. Oral Sci..

[43] U.S. Census Bureau QuickFacts: Lowa. https://www.census.gov/quickfacts/IA.

[44] Hu Z., Artibani M., Alsaadi A., Wietek N., Morotti M., Shi T., Zhong Z., Gonzalez L.S., El-Sahhar S., Carrami E.M. (2020). The Repertoire of Serous Ovarian Cancer Non-Genetic Heterogeneity Revealed by Single-Cell Sequencing of Normal Fallopian Tube Epithelial Cells. Cancer Cell.

[45] Miller M.D., Salinas E.A., Newtson A.M., Sharma D., Keeney M.E., Warrier A., Smith B.J., Bender D.P., Goodheart M.J., Thiel K.W. (2019). An Integrated Prediction Model of Recurrence in Endometrial Endometrioid Cancers. Cancer Manag. Res..

[46] Liao Y., Smyth G.K., Shi W. (2013). The Subread Aligner: Fast, Accurate and Scalable Read Mapping by Seed-and-Vote. Nucleic Acids Res..

[47] Liao Y., Smyth G.K., Shi W. (2014). FeatureCounts: An Efficient General Purpose Program for Assigning Sequence Reads to Genomic Features. Bioinformatics.

[48] Love M.I., Huber W., Anders S. (2014). Moderated Estimation of Fold Change and Dispersion for RNA-Seq Data with DESeq2. Genome Biol..

[49] Aran D., Looney A.P., Liu L., Wu E., Fong V., Hsu A., Chak S., Naikawadi R.P., Wolters P.J., Abate A.R. (2019). Reference-Based Analysis of Lung Single-Cell Sequencing Reveals a Transitional Profibrotic Macrophage. Nat. Immunol..

[50] Hao Y., Stuart T., Kowalski M.H., Choudhary S., Hoffman P., Hartman A., Srivastava A., Molla G., Madad S., Fernandez-Granda C. (2024). Dictionary Learning for Integrative, Multimodal and Scalable Single-Cell Analysis. Nat. Biotechnol..

[51] Robin X., Turck N., Hainard A., Tiberti N., Lisacek F., Sanchez J.C., Müller M. (2011). PROC: An Open-Source Package for R and S+ to Analyze and Compare ROC Curves. BMC Bioinform..

[52] Leek J.T., Johnson W.E., Parker H.S., Jaffe A.E., Storey J.D. (2012). The SVA Package for Removing Batch Effects and Other Unwanted Variation in High-Throughput Experiments. Bioinformatics.

[53] Harris M.A., Clark J., Ireland A., Lomax J., Ashburner M., Foulger R., Eilbeck K., Lewis S., Marshall B., Mungall C. (2004). The Gene Oncology (GO) Database and Informatics Resource. Nucleic Acids Res..

[54] R Core Team (2019). R: A Language and Environment for Statistical Computing.

