# Supplementary material for: scRNA-seq Can Identify Different Cell Populations in Ovarian Cancer Bulk RNA-seq Experiments
Source: Int J Mol Sci. 2025 Aug 4;26(15):7512. doi: 10.3390/ijms26157512 (PMC12347332; doi:10.3390/ijms26157512)
Supplement: Supplementary file 1 [file ijms-26-07512-s001.zip › Supplementary RNA-seq deconvolution review FINAL 7-23-25.pdf]

TITLE:

scRNA-seq can identify different cell populations in ovarian cancer bulk RNA-seq experiments

Authors:

Sofia Gabrilovich, MD[1], Eric Devor, PhD[2], Nicholas Cardillo, MD[3], David Bender, MD[2], Michael Goodheart, MD[2], Jesus Gonzalez-Bosquet, MD, PhD[2]

[1] Medical College of Wisconsin, Milwaukee, WI, USA

[2] University of Iowa Carver College of Medicine, Iowa City, IA, USA

[3] Jefferson Health, Philadelphia, PA, USA

Corresponding Author:

Jesus Gonzalez-Bosquet, MD, PhD

Email: jesus-gonzalezbosquet@uiowa.edu

Keywords: Ovarian cancer, scRNA sequencing, deconvolution

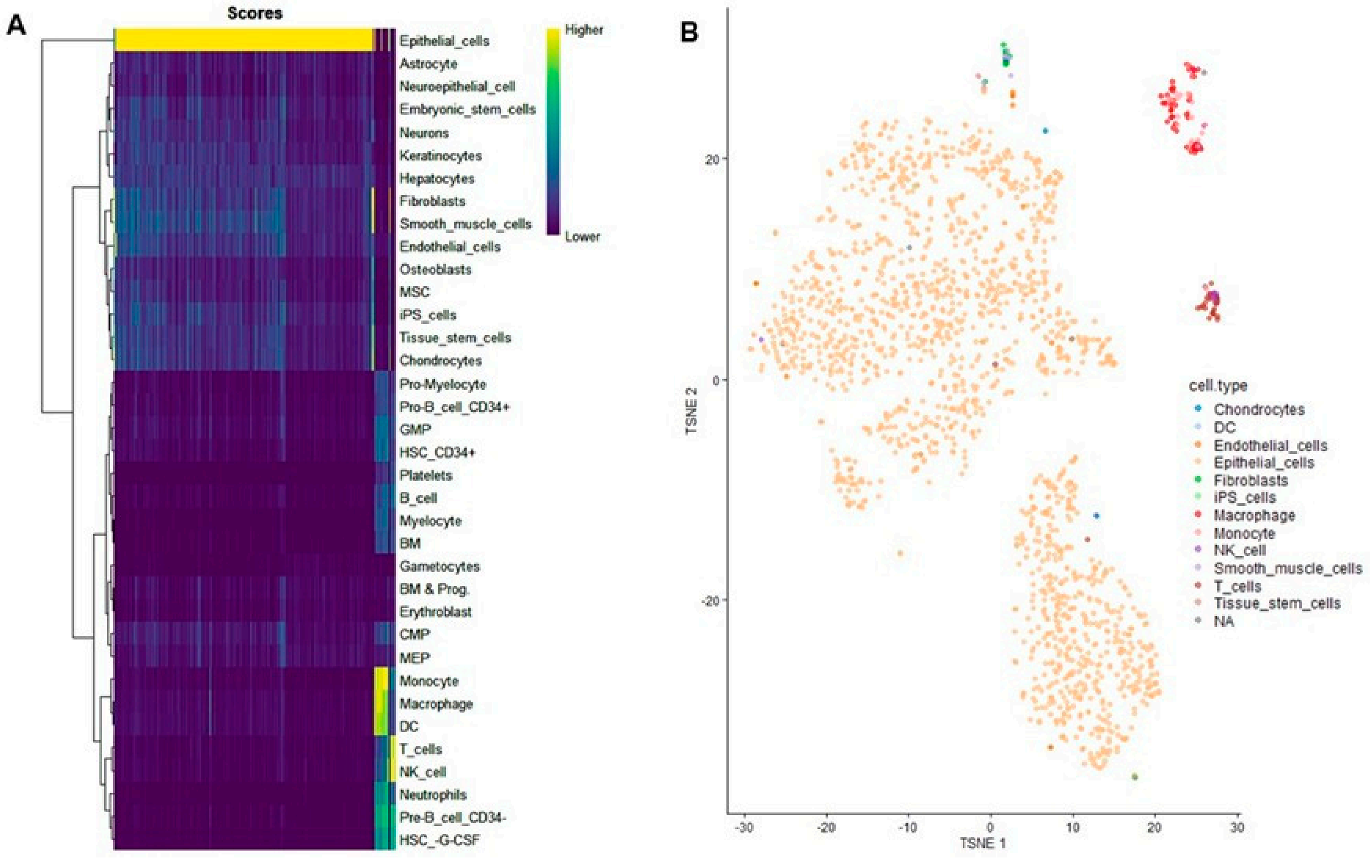

Supplementary Figure S1: Annotation of cells included in the GSE139079 scRNAseq experiment using *SingleR* package.

A. We annotated all cells against built references from the Human Primary Cell Atlas (HPCA). Heatmap based on the matrix of gene log-expression values for different cell types.

B. Dimensionality reduction with t-SNE plotted using the *plotReducedDim* function. Each point represents a cell and is colored according to its cell type label based on the HPCA reference. The fallopian tube scRNAseq experiment shows a majority of epithelial cells (over 90%), few (7%) immune cells, and 0.2% of tissue specific stem cells (iPs_cells and Tissue_stem_cells).

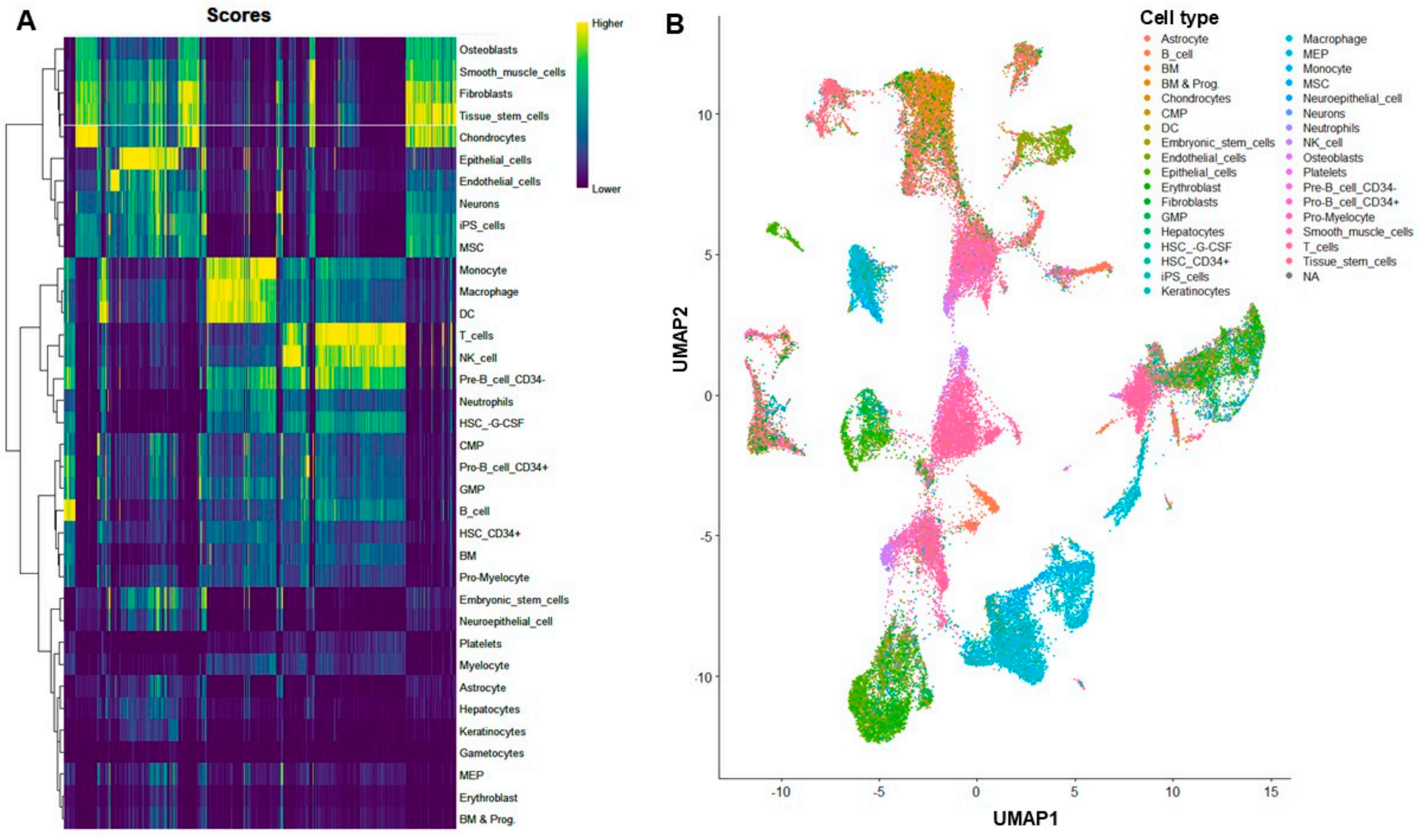

Supplementary Figure S2: Annotation of cells included in the GSE189955 scRNAseq experiment using *SingleR* package.
A. We annotated all cells against built references from the Human Primary Cell Atlas (HPCA). Heatmap based on the matrix of gene log-expression values for different cell types.
B. Dimensionality reduction with UMAP plotted by cell type and the *DimPlot* function. Each point represents a cell and is colored according to its cell type label based on the HPCA reference. The GSE154600 scRNAseq with HGSC tumor cells and annotated with HPCA as reference shows a mix of epithelial cells, immune cells, and connective tissue cells.

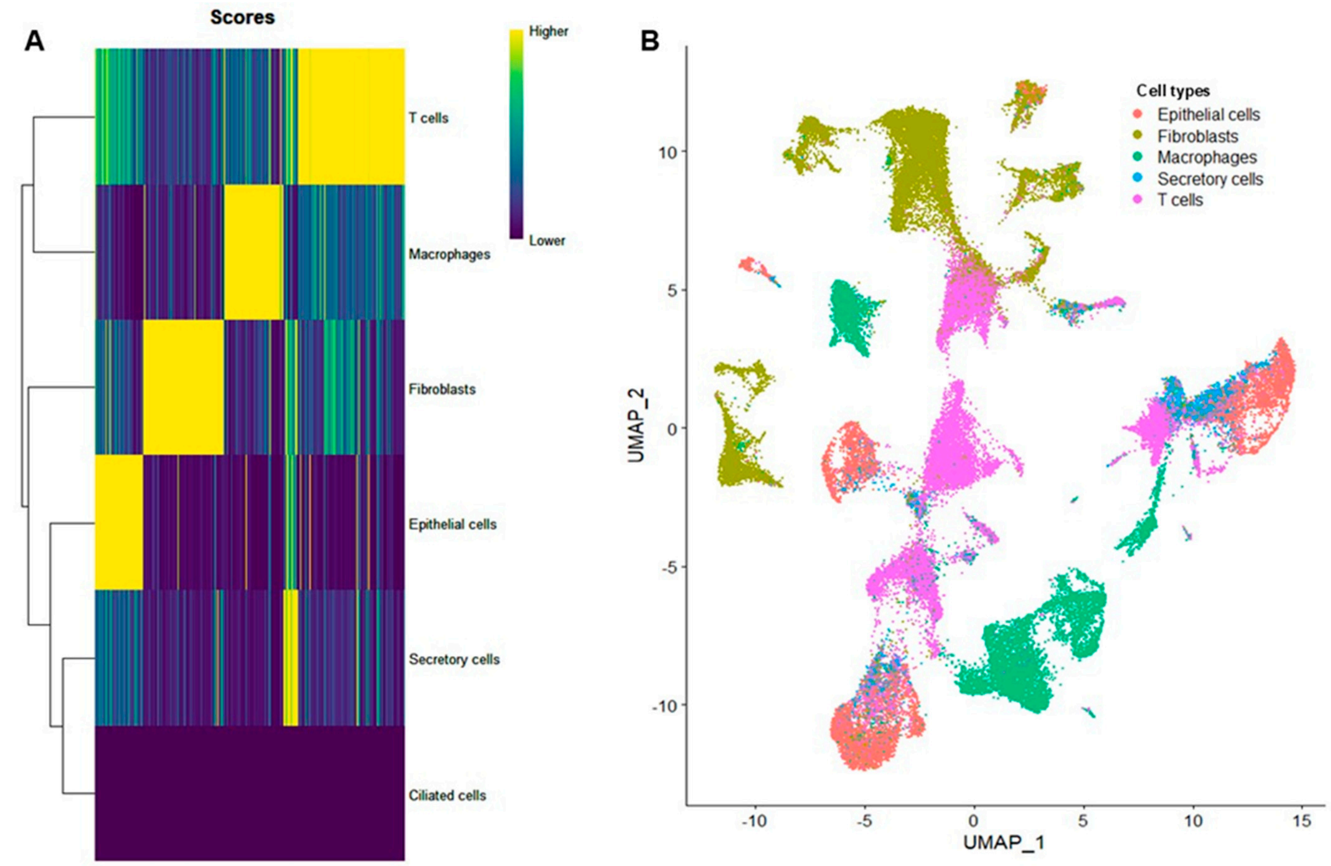

Supplementary Figure S3: Annotation of cells included in the GSE154600 scRNAseq experiment using *SingleR* package.
A. We annotated all cells against built references from the GSE154600 scRNAseq experiment. Heatmap based on the matrix of gene log-expression values for different cell types .
B. Dimensionality reduction with UMAP plotted by cell type and the *DimPlot* function. Each point represents a cell and is colored according to its cell type label based on the GSE189955 dataset. The GSE154600 scRNAseq with HGSC tumor cells and annotated with the GSE189955 dataset shows a mix of epithelial cells, secretory cells and immune cells, with absence of ciliated cells.

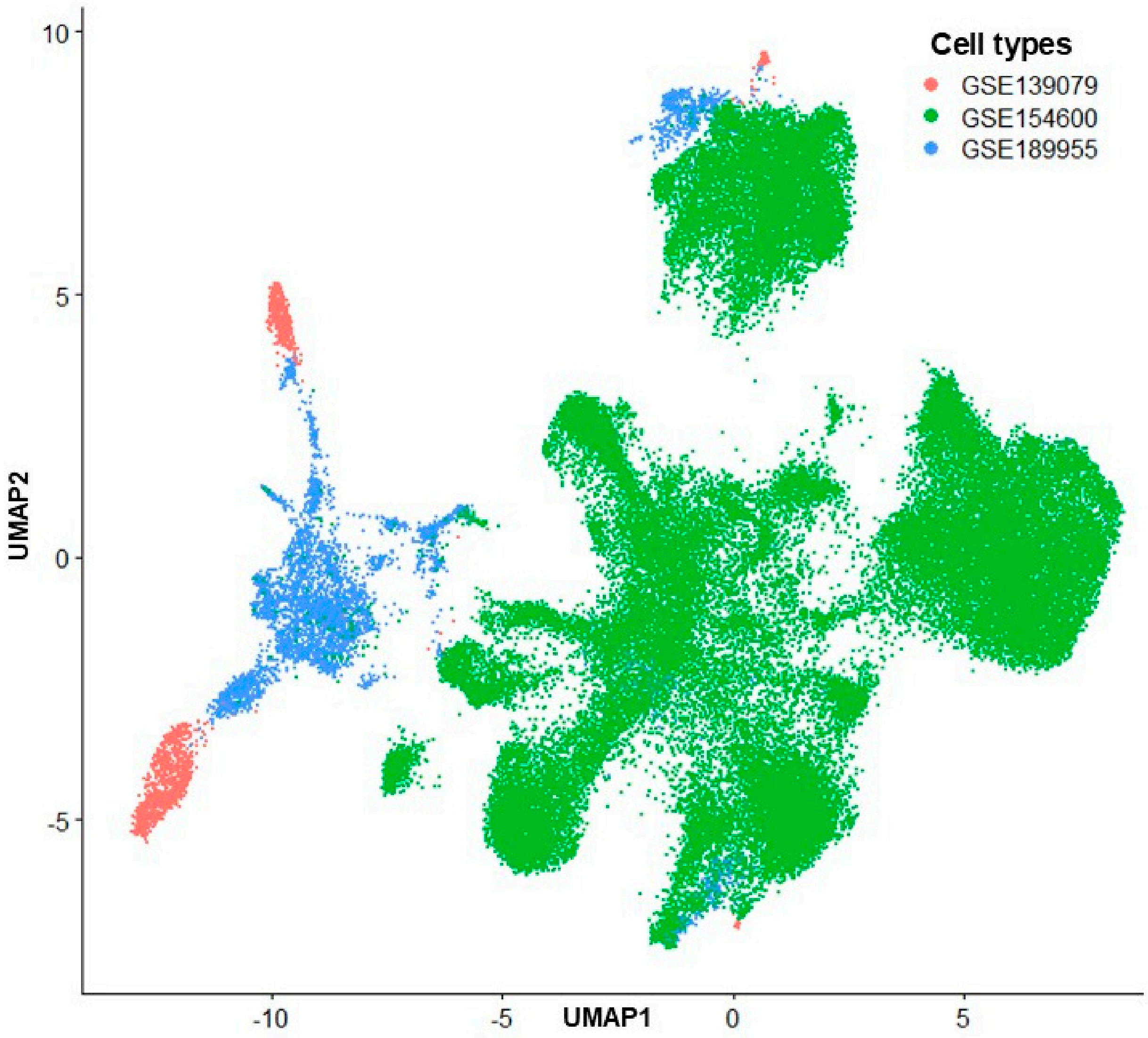

Supplementary Figure S4: Integration of all reference scRNAseq experiments with the Seurat package for posterior deconvolution.

The database extracted from the bioproject GSE189955 was annotated by the authors themselves. We integrated datasets extracted from the GSE189955, GSE139079, and GSE154600 datasets. The integrated database included more than 59,000 ovarian cancer and fallopian tube cells with more than 74,000 gene expression features. We annotated all cells of the integrated dataset based on the initial bioproject.

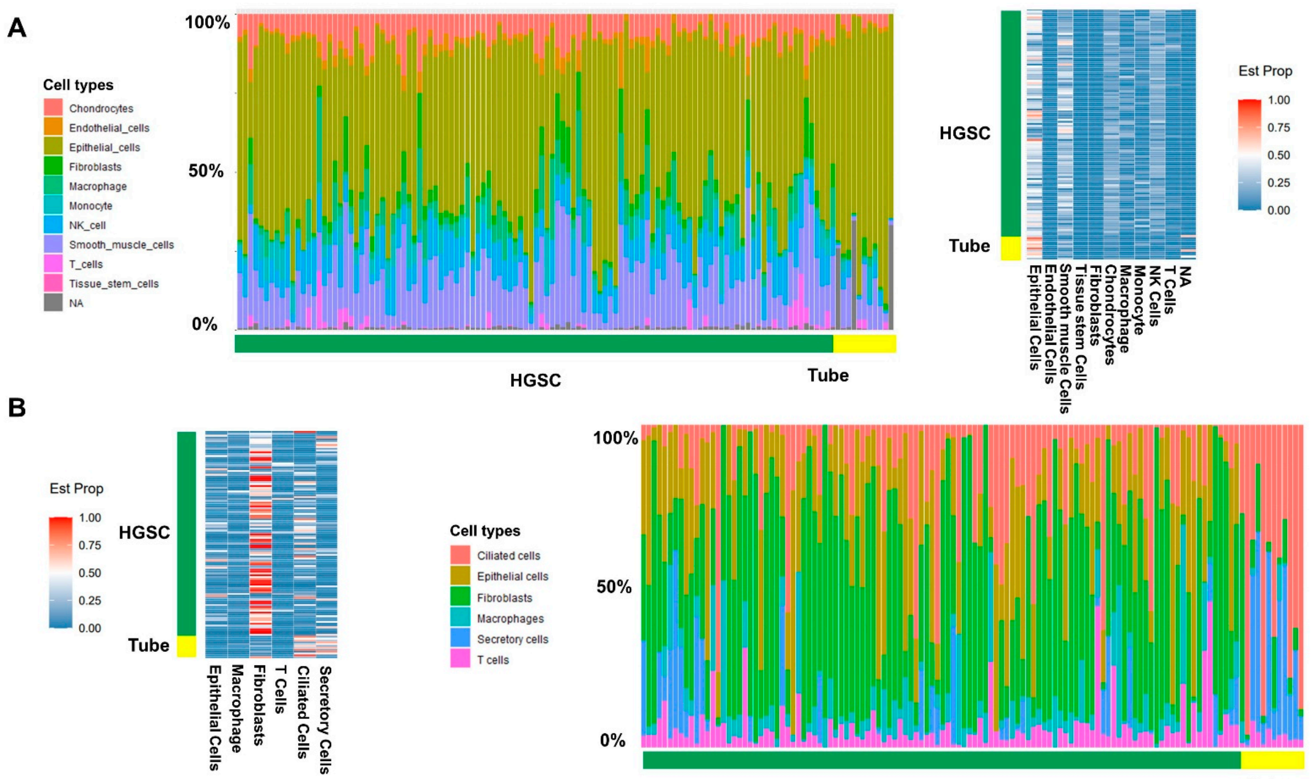

Supplementary Figure S5: Deconvolution of bulk UI RNAseq experiments with patterns from different scRNAseq as reference (using *MuSiC*).

A. Using normal fallopian tube scRNAseq dataset (GSE139079) as reference annotated with HPCA cell types: Left panel: Patient-based bar plot of percentage of cell types by each patient sample (horizontal) of the UI dataset; color coding at the left. Right panel: Cell-based heatmap of estimated proportion for each sample (horizontal). For the deconvolution analysis of UI specimens, we include both HGSC and normal tubal samples. Epithelial cells are more predominant in the normal tube of the UI cohort, while the HGSC presents more immune and connective tissue cells.

B. Using HGSC scRNAseq dataset (GSE189955) as reference. Left panel: Cell-based heatmap of estimated proportion for each sample. For the deconvolution analysis of UI specimens, we include both HGSC and normal tubal samples. Right panel: Patient-based bar plot of percentage of cell types of the UI dataset; color coding at the left. Using the HGSC scRNAseq dataset as a reference, there is a higher estimation of fibroblast in the bulk RNAseq experiment, except for the normal tubal cells, where there is a predominance of ciliated and secretory cells.

.

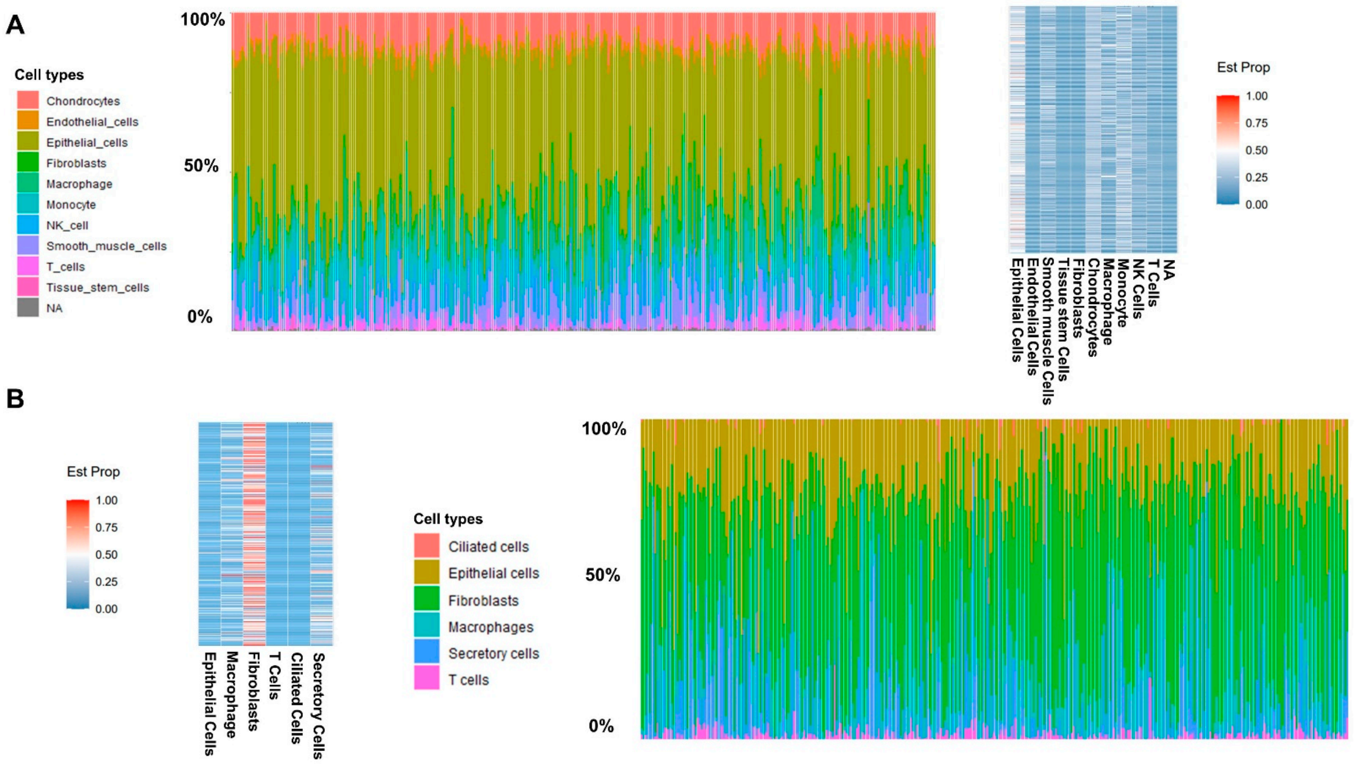

Supplementary Figure S6: Deconvolution of bulk RNAseq from TCGA with patterns from different scRNAseq as reference (using *MuSiC*).

A. Using normal fallopian tube scRNAseq dataset (GSE139079) as reference annotated with HPCA cell types: Left panel: Patient-based bar plot of percentage of cell types of the HGSC TCGA dataset; color coding at the left. Right panel: Cell-based heatmap of estimated proportion for each sample. There are only HGSC samples in TCGA datasets. Epithelial cells are predominant in the TCGA set of samples.

B. Using HGSC scRNAseq dataset (GSE189955) as reference. Left panel: Cell-based heatmap of estimated proportion for each sample. Right panel: Patient-based bar plot of percentage of cell types; color coding at the left. Using the HGSC scRNAseq dataset as a reference, there is a higher estimation of fibroblast in the bulk RNAseq experiment, similarly to the UI bulk RNAseq convolution and cell type estimation.

A.

	Cell proportions			
	HGSC	Tube	OR	p-value
Chondrocytes	0.08	0.03	5.4×10^{21}	**<0.001***
Endothelial cells	0.02	0.01	1.3×10^{49}	**0.010***
Epithelial cells	0.49	0.70	2×10^{-5}	**<0.001***
Fibroblasts	0.04	0.02	1.6×10^{8}	0.170
Macrophage	0.06	0.02	8.6×10^{12}	0.051
Monocyte	0.03	0.02	1.7×10^{5}	0.308
No classified	0.01	0.08	3.9×10^{-9}	0.065
NK cells	0.10	0.03	3.9×10^{25}	**<0.001***
Smooth muscle cells	0.17	0.09	3.9×10^{5}	**0.010***
T cells	0.01	0.01	1.7×10^{4}	0.556
Tissue stem cells	0.00	0.00	0.00	0.990

	OR	p-value
Chondrocytes	2.5×10^{20}	**0.020***
Endothelial cells	3.3×10^{14}	0.469
Epithelial cells	2.8×10^{-4}	0.202
NK cells	5.9×10^{30}	**0.003***
Smooth muscle cells	1.4×10^{-7}	0.176

B.

	Cell proportions			
	HGSC	Tube	OR	p-value
Ciliated cells	0.14	0.55	1.7×10^{-3}	**<0.001***
Epithelial cells	0.23	0.00	5×10^{93}	0.2938
Fibroblasts	0.44	0.13	3.8×10^{2}	**0.003***
Macrophages	0.07	0.02	7.6×10^{10}	**0.046***
Secretory cells	0.05	0.27	5.3×10^{-4}	**<0.001***
T cells	0.07	0.04	1.5×10^{8}	0.1250

	OR	p-value
Ciliated cells	0.02	**0.020***
Fibroblasts	17.8	0.2164
Macrophages	2.7×10^{4}	0.2891
Secretory cells	1.1×10^{-22}	**0.027***

Supplementary Figure S7: Association analysis of cell proportions with ovarian cancer in the UI dataset after deconvolution.

A. When comparing the proportion of cell types between HGSC and normal FT after deconvolution with HPCA as reference, in the univariate analysis (upper panel) there was a decrease in epithelial cells in HGSC, with increases in NK cells, and other types of connective tissue cells: chondrocytes, endothelial, smooth muscle cells. In the multivariate analysis (lower panel) NK cells and chondrocytes were increased in HGSC after accounting for the other cell types.

B. When comparing the proportion of cell types between HGSC and normal FT after deconvolution with GSE189955 as reference, in the univariate analysis (upper panel) there was a decrease in ciliated and secretory cells in malignant tumors, and an increase in fibroblasts and macrophages. In the multivariate analysis (lower panel) ciliated and secretory cells persistently decreased in HGSC after accounting for the other cell types.

*Statistically significant (p-value <0.05) (OR= odds ratio)

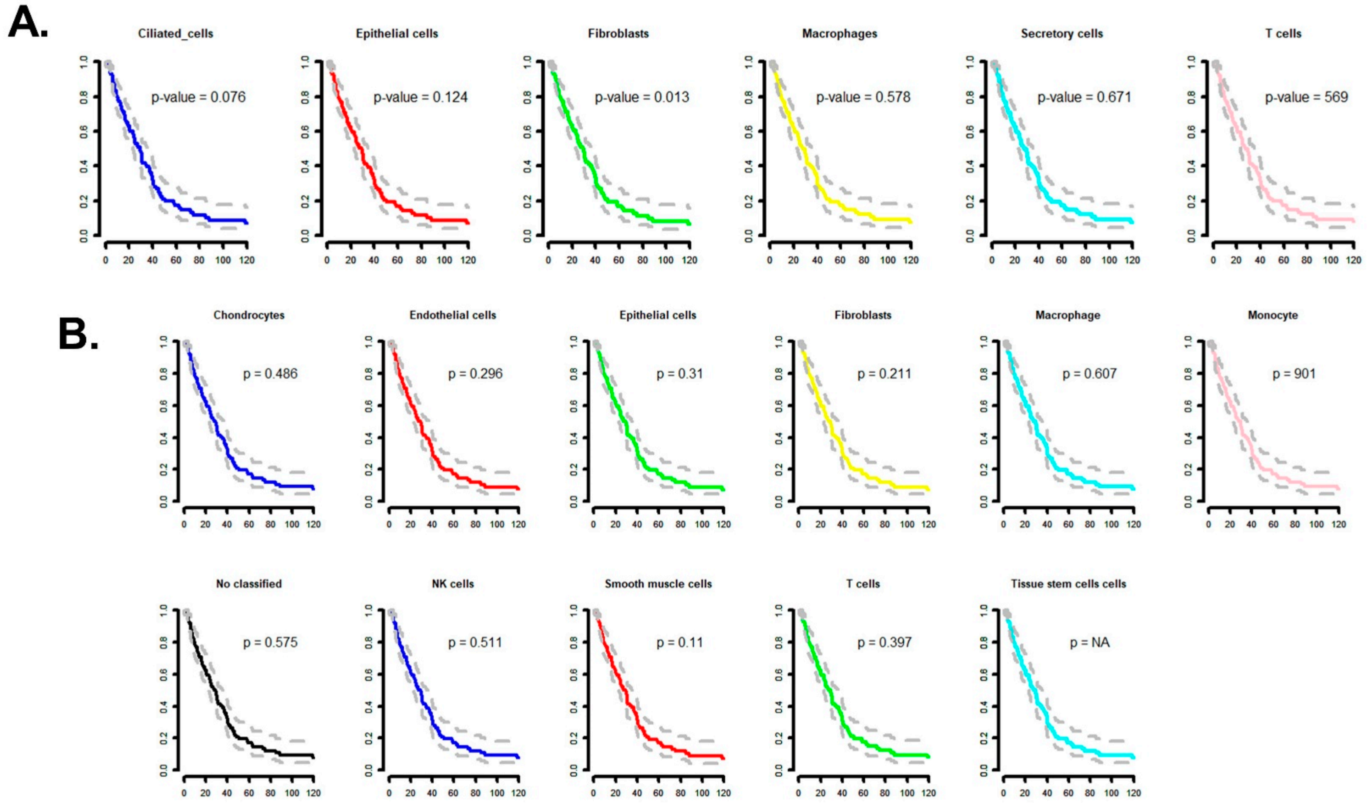

Supplementary Figure S8: Association analysis with survival in the UI dataset by cellular type after deconvolution with GSE189955 or with GSE139079 annotated with HPCA as respective references.
The *coxph* function of the R package *survival* was used for survival analysis. P-value was assessed by the log-rank test and survival curves were plotted with the *survfit* (*plot(survfit(cox_model)*) as recommended by the authors of the package (https://cran.r-project.org/web/packages/survival/vignettes/survival.pdf). KM curves were represented by the median survival and 95% confidence intervals.
A. Fibroblasts werethe only cell type that were significantly negatively associated with survival (p=0.013, HR: 2.6), after the deconvolution with GSE189955 as reference. HR: hazard ratio; low and high are the 95% CI of the HR. In the univariate analysis of clinical features associated with survival (including all variables from Table 1), age at diagnosis, Charlson co-morbidity index, residual disease after surgery, response to chemotherapy, and neoadjuvant chemotherapy were significant (p<0.05). After adjusting for all these factors, age at diagnosis (p=0.046) and response to chemotherapy (p=$3x10^{-7}$) remained independently significant. Fibroblasts were no longer significant in this multivariate model (p= 0.189)
B. No cell type that was significantly associated with survival after the deconvolution with GSE139079 annotated with HPCA as reference.

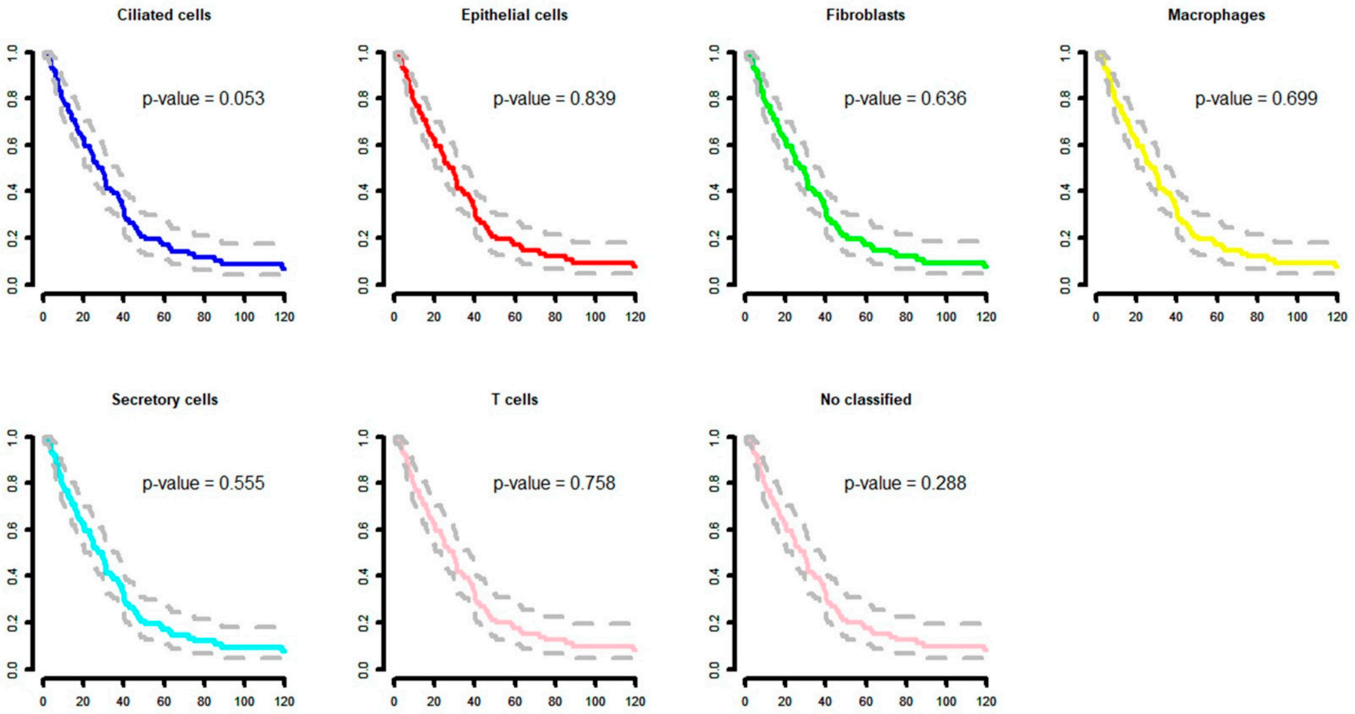

Supplementary Figure S9: Association analysis with survival in the UI dataset by cellular type after deconvolution with the integrated set as reference. Ciliated cells were the only cell type that trended towards significance of a negative association with survival (p=0.053), after the deconvolution with integrated set as reference. HR: hazard ratio; low and high are the 95% CI of the HR. In the univariate analysis of clinical features associated with survival (including all variables from Table 1), age at diagnosis, Charlson co-morbidity index, residual disease after surgery, response to chemotherapy, and neoadjuvant chemotherapy were significant ($p<0.05$). After adjusting for all these factors, age at diagnosis (p=0.046) and response to chemotherapy ($p=3x10^{-7}$) remained independently significant. The proportion of ciliated cells was not significant when added to this multivariate model (p= 0.338).
Survival analysis was performed as before.

Supplementary Table S1: Association analysis of cell proportions with response to chemotherapy and optimal surgical debulking in the UI dataset.
After deconvolution with the integrated database as reference no cellular type was associated with response to chemotherapy. Only macrophages were close to this association with chemo-response (p=0.139). OR: odds ratio.

Response to chemotherapy

	Responders	Non-Responders	OR	p-value
**Ciliated cells**	0.04	0.03	$2.9x10^{-5}$	0.171
**Epithelial cells**	0.28	0.30	1.23	0.802
**Fibroblasts**	0.48	0.51	1.34	0.653
**Macrophages**	0.05	0.01	$9.1x10^{-5}$	0.139
**No classification**	0.05	0.05	0.006	0.596
**Secretory cells**	0.08	0.09	2.43	0.640
**T cells**	0.01	0.01	2.59	0.838

Optimal surgery

	Optimal	Suboptimal	OR	p-value
**Ciliated cells**	0.04	0.03	0.002	0.363
**Epithelial cells**	0.32	0.25	0.37	0.192
**Fibroblasts**	0.45	0.55	2.23	0.171
**Macrophages**	0.05	0.02	0.10	0.286
**No classification**	0.05	0.05	$6.1x10^{-7}$	0.117
**Secretory cells**	0.09	0.09	1.06	0.974
**T cells**	0.00	0.02	$5.31x10^{4}$	0.236

Supplementary Table S2: Association analysis of cell proportions with response to chemotherapy and optimal surgical debulking in the UI dataset.
After deconvolution with HPCA annotation as reference, chondrocytes (p=0.033), macrophages (p=0.035), and smooth muscle cells (p=0.033) were associated with response to chemotherapy in the multivariate analysis, even after adjusting for neoadjuvant chemotherapy (p-0.014) and residual disease (p=0.033). In the univariate analysis of clinical features associated with optimal surgery, only disease in the chest (p=0.067) and proportion of macrophages (p=0.061) were close to be significant.
*Statistically significant (p-value <0.05). OR: odds ratio.

**Response to chemotherapy**			**Univariate**		**Multivariate**	
	**Responders**	**Non-Responders**	**OR**	**p-value**	**OR**	**p-value**
**Chondrocytes**	0.08	0.07	3×10^{-5}	0.078	7×10^{-8}	**0.033***
**Endothelial cells**	0.02	0.03	5.8×10^{2}	0.500	NS	
**Epithelial cells**	0.48	0.50	2.08	0.531	NS	
**Fibroblasts**	0.04	0.04	0.20	0.752	NS	
**Macrophages**	0.07	0.04	0.001	0.094	2.8×10^{-5}	**0.035***
**Monocytes**	0.03	0.02	6×10^{-4}	0.219	NS	
**No classification**	0.01	0.01	1.1×10^{-6}	0.733	NS	
**NK cells**	0.10	0.10	1.96	0.907	NS	
**Smooth muscle cells**	0.16	0.19	43.61	0.126	1.3×10^{3}	**0.033***
**T cells**	0.01	0.01	1.7×10^{4}	0.556	NS	
**Tissue stem cells**	0.00	8.3×10^{-5}	0.00	0.990	NS	
**Clinical features**						
**Age**			1.05	**0.011***	1.04	0.055
**BMI**			1.05	0.160	NS	
**Charlson Index**			0.11	0.068	0.461	0.564
**Preop CA125**			1.00	0.874	NS	
**Disease upper abdomen**			2.28	0.076	1.194	0.232
**Disease in chest**			2.7×10^{-8}	0.991	NS	
**Neoadjuvant chemotherapy**			8.39	**0.009***	10.21	**0.014***
**FIGO Stage**			2.67	0.069	1.849	0.065
**Optimal surgery (R0+R1)**			0.46	0.097	0.406	0.172
**Residual micro (R0)**			0.26	**0.048***	0.14	**0.033***
**Surgical Complexity score**			2.7×10^{-8}	0.990	NS	

**Optimal surgery**			**Univariate**		**Multivariate**	
	**Optimal**	**Suboptimal**	**OR**	**p-value**	**OR**	**p-value**
**Chondrocytes**	0.08	0.07	0.06	0.590	NS	
**Endothelial cells**	0.03	0.02	6×10^{-7}	0.226	NS	
**Epithelial cells**	0.47	0.51	2.97	0.327	NS	
**Fibroblasts**	0.04	0.03	0.01	0.353	NS	
**Macrophage**	0.07	0.04	0.001	0.061	0.002	0.111
**Monocyte**	0.03	0.03	0.12	0.693	NS	
**none**	0.01	0.01	0.001	0.855	NS	

**NK cells**	0.10	0.10	29.99	0.529	NS	
**Smooth muscle cells**	0.17	0.18	3.11	0.597	NS	
**T cells**	0.01	0.01	0.001	0.388	NS	
**Tissue stem cells**	0.00	0.00	0	NA	NS	
**Clinical features**						
**Age**			0.99	0.587	NS	
**BMI**			1.01	0.638	NS	
**Charlson Index**			1.06	0.946	NS	
**Preop CA125**			1.00	0.572	NS	
**Disease upper abdomen**			1.27	0.564	NS	
**Disease in chest**			4.79	0.069	$2x10^{-8}$	0.991
**Neoadjuvant chemotherapy**			8.39	0.558	NS	
**FIGO Stage**			$7.7x10^{6}$	0.989	NS	
**Optimal surgery (R0+R1)**			$8.4x10^{-24}$	0.999	NS	
**Residual micro (R0)**			$1x10^{-8}$	0.990	NS	
**Surgical Complexity score**			0.53	0.508	NS	

Supplementary Table S3: Clinical characteristics in TCGA HGSC dataset.

		TCGA (N=352)
**Preoperative characteristics**	**Age (mean)**	59
	**Neoadjuvant chemotherapy**	1
**Operative characteristics**	**FIGO Stage**	
	**I**	1
	**II**	20
	**III**	272
	**IV**	53
	**N/A**	2
**Outcomes**	**Surgical outcomes***	
	**Optimal**	180
	**Suboptimal**	62
	**Response to chemotherapy****	
	**Yes**	175
	**No**	85

*Surgical outcomes were classified as no residual disease greater than 1cm in size (R1 or also known as 'optimal cytoreduction'), and suboptimal debulking, or residual disease of more than 1 cm (or R2).
**Response to chemotherapy: Patients that responded to chemotherapy are those that had no recurrence for at least 6 months after finishing treatment. Patients that did not respond are those that progressed during treatment (refractory to therapy), tumor was not modified significantly by treatment, or progress within 6 months after finishing treatment (resistant to therapy).

Supplementary Table S4: Association analysis of cell proportions with response to chemotherapy and optimal surgical debulking in the TCGA dataset.
A. After deconvolution with the integrated database as reference, in the univariate analysis fibroblasts (p=0.010) were associated with non-response to chemotherapy, and epithelial cells (p=0.031) were associated with response to chemotherapy. According to the univariate analysis, the clinical features associated with response to chemotherapy, or risk of disease relapse, were stage and residual disease after surgery (p<0.05). After adjusting for all these factors, residual disease after surgery (p=0.001) remained significant. There was no significant difference in the number of fibroblasts or epithelial cells in this multivariate model.
B. After deconvolution with the integrated database as reference, in the univariate analysis fibroblasts (p=0.037) were associated with optimal surgery, even after adjusting for significant clinical variables (response to chemotherapy, p<0.001) in the multivariate analysis.
*Statistically significant (p-value <0.05). OR: odds ratio.

**A. Response to chemotherapy**			**Univariate**		**Multivariate**	
	**Responders**	**Non-Responders**	**OR**	**p-value**	**OR**	**p-value**
**Ciliated cells**	0.002	0.001	$1.3x10^{31}$	0.115	$2x10^{16}$	0.525
**Epithelial cells**	0.72	0.67	4.34	**0.031***	0.76	0.817
**Fibroblasts**	0.10	0.15	0.09	**0.010***	0.33	0.478
**Macrophages**	0.11	0.12	0.69	0.635	NS	
**No classification**	0.03	0.03	$3.1x10^{4}$	0.287	NS	
**Secretory cells**	0.04	0.04	1.91	0.823	NS	
**Clinical features**						
**Age**			0.996	0.791	NS	
**Neoadjuvant chemotherapy**			$1x10^{6}$	0.987	NS	
**Stage**			0.12	**0.045***	0.99	0.982
**Optimal surgery**			0.25	**<0.001***	0.27	**0.001***

**B. Optimal surgery**			**Univariate**		**Multivariate**	
	**Optimal**	**Suboptimal**	**OR**	**p-value**	**OR**	**p-value**
**Ciliated cells**	0.002	0.001	$1.2x10^{19}$	0.344	NS	
**Epithelial cells**	0.72	0.68	0.31	0.130	0.56	0.666
**Fibroblasts**	0.11	0.15	8.57	**0.037**	13.402	**0.044***
**Macrophages**	0.11	0.10	0.90	0.914	NS	
**No classification**	0.03	0.03	0.009	0.656	NS	
**Secretory cells**	0.04	0.04	4.55	0.625	NS	
**Clinical features**						
**Age**			1.03	0.047	1.02	0.287
**Neoadjuvant chemotherapy**			$1x10^{6}$	0.987	NS	
**Stage**			2.01	0.058	1.53	0.387
**Response to chemotherapy**			0.25	**<0.001***	0.24	**<0.001***

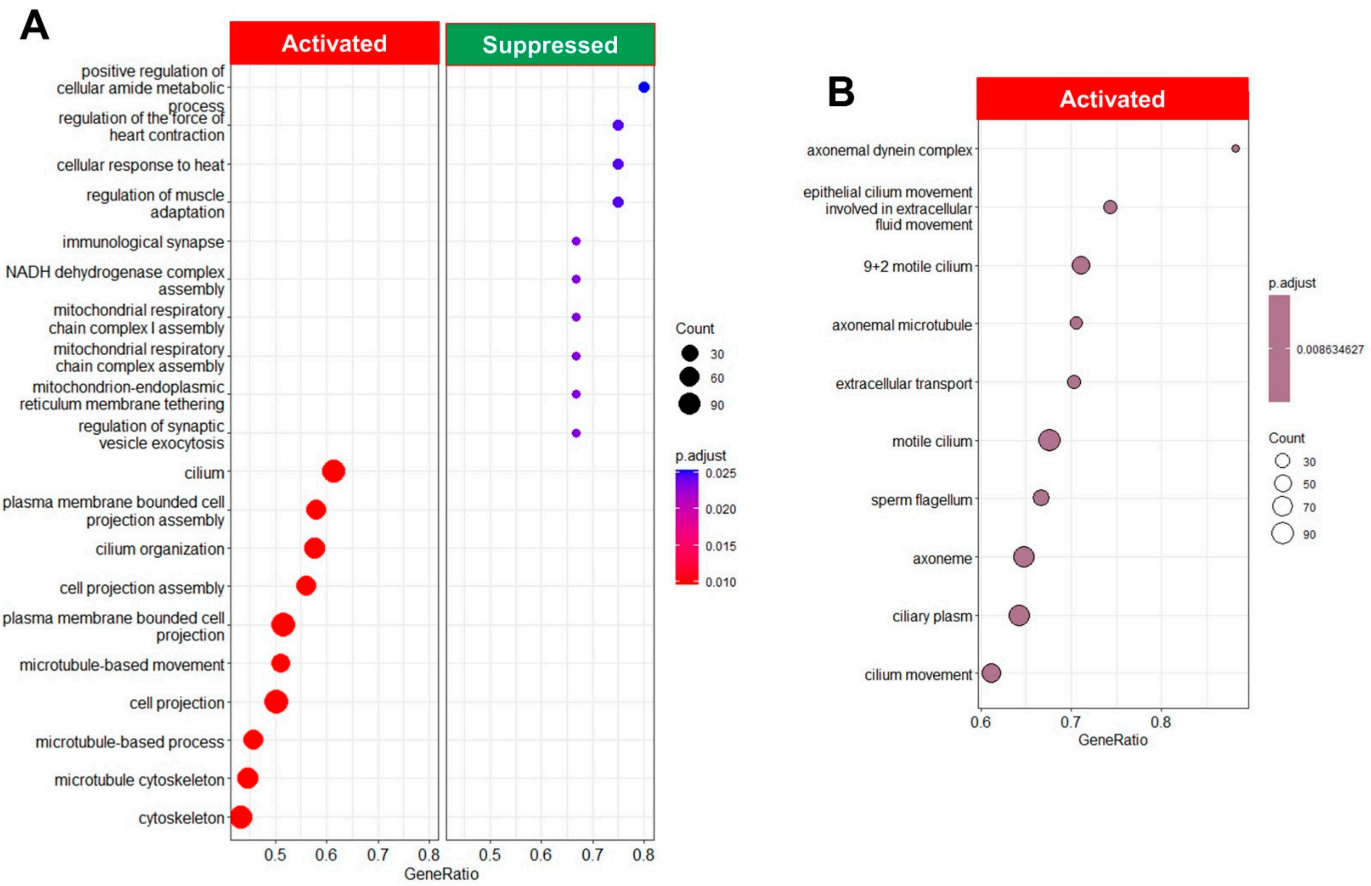

Supplementary Figure S10: Pathway association analysis associated with ciliated cells using GO database with *clusterProfiler.*

A. Pathway enrichment analysis using the characteristic genes resulting on the annotation of ciliated cells ($p<10^{-4}$) derived from GSE189955 dataset.

B. Same pathway analysis but resulting from characteristic gene expression from the integrated dataset: GSE139079, GSE189955, and GSE154600.

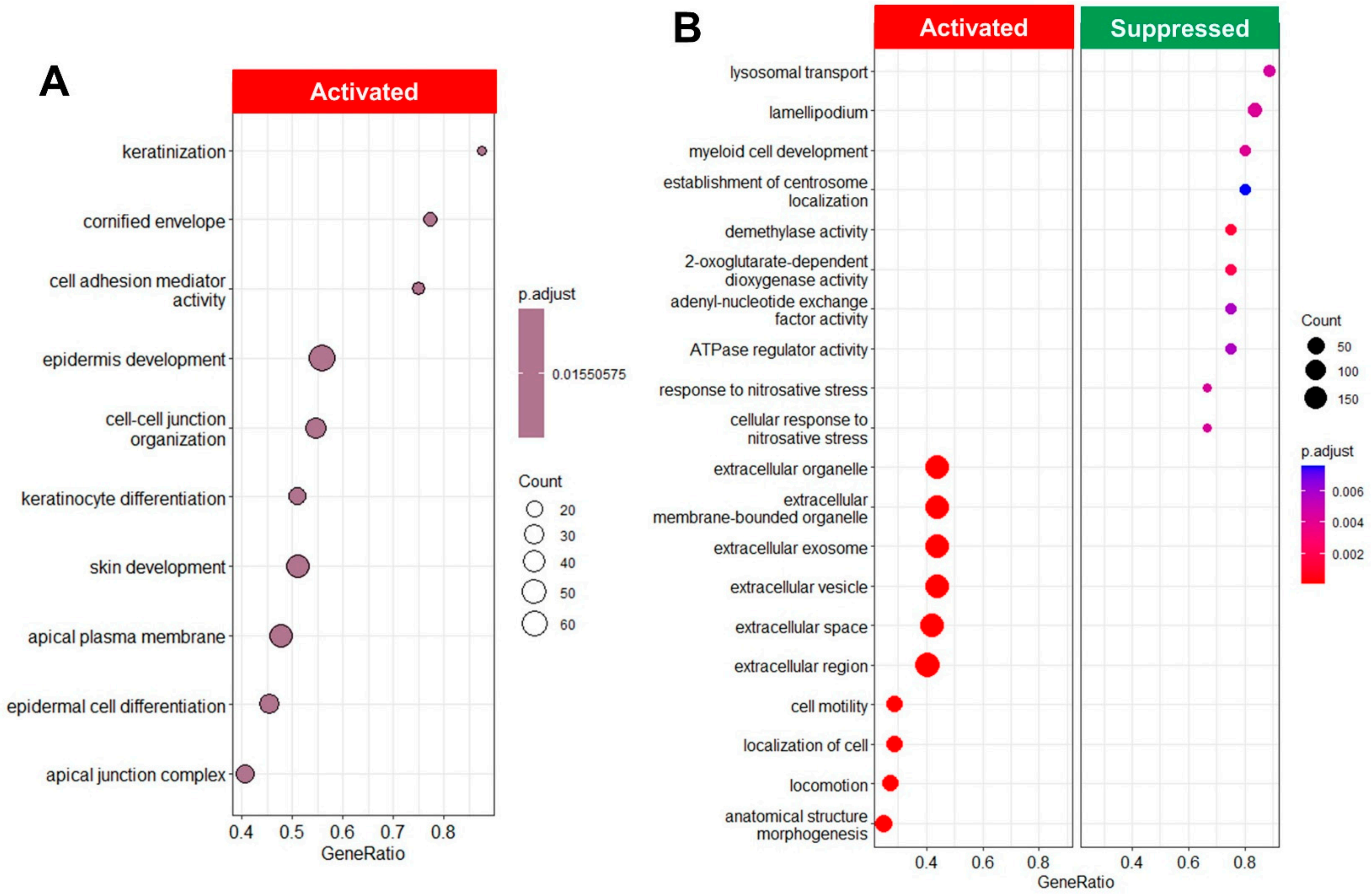

Supplementary Figure S11: Pathway association analysis associated with epithelial cells using GO database.
A. Pathway enrichment analysis using the characteristic genes resulting on the annotation of epithelial cells ($p<10^{-4}$) derived from GSE189955 dataset.
B. Same pathway analysis but resulting from characteristic gene expression from the integrated dataset.

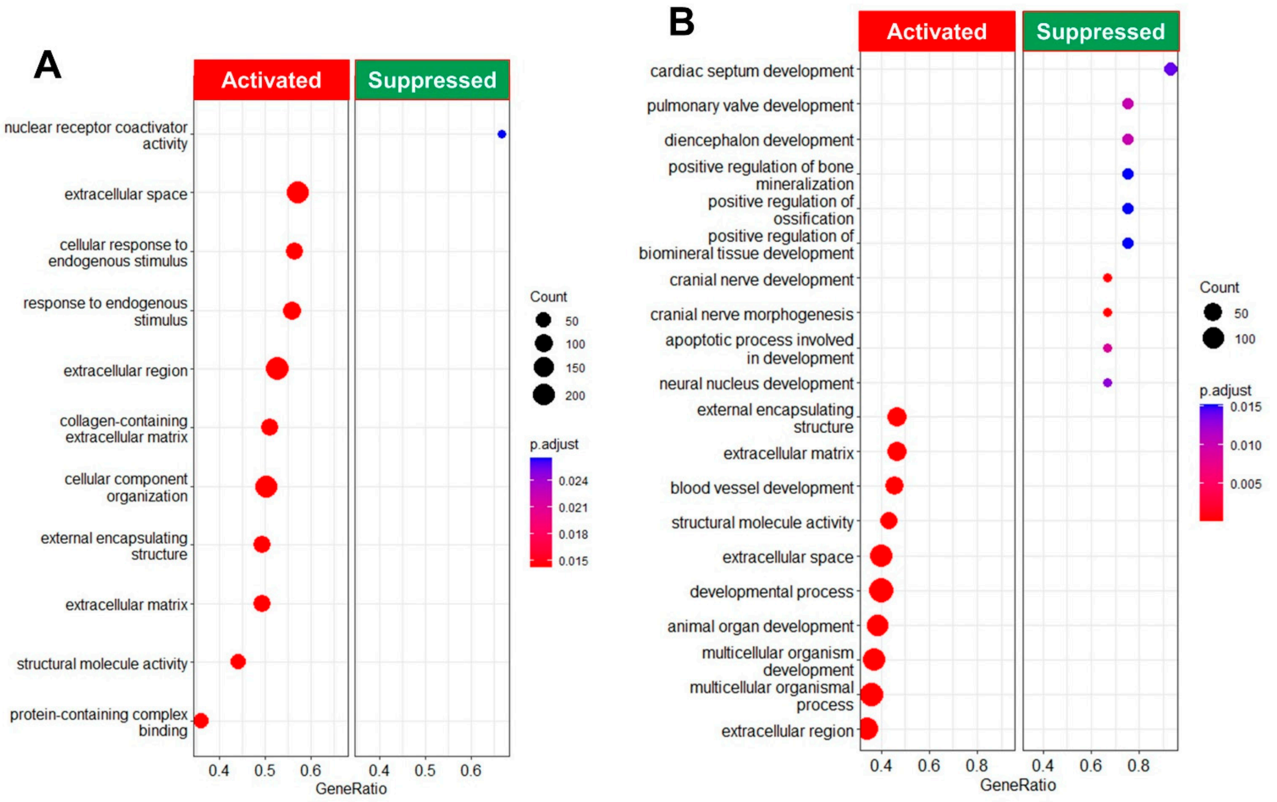

Supplementary Figure S12: Pathway association analysis associated with fibroblasts using GO database.
A. Pathway enrichment analysis using the characteristic genes resulting on the annotation of fibroblasts ($p<10^{-4}$) derived from GSE189955 dataset.
B. Same pathway analysis but resulting from characteristic gene expression from the integrated dataset.
GeneRatio: gene abundance within macrophages versus non-macrophages. *Count*: number of significant genes participating in the GO pathway.

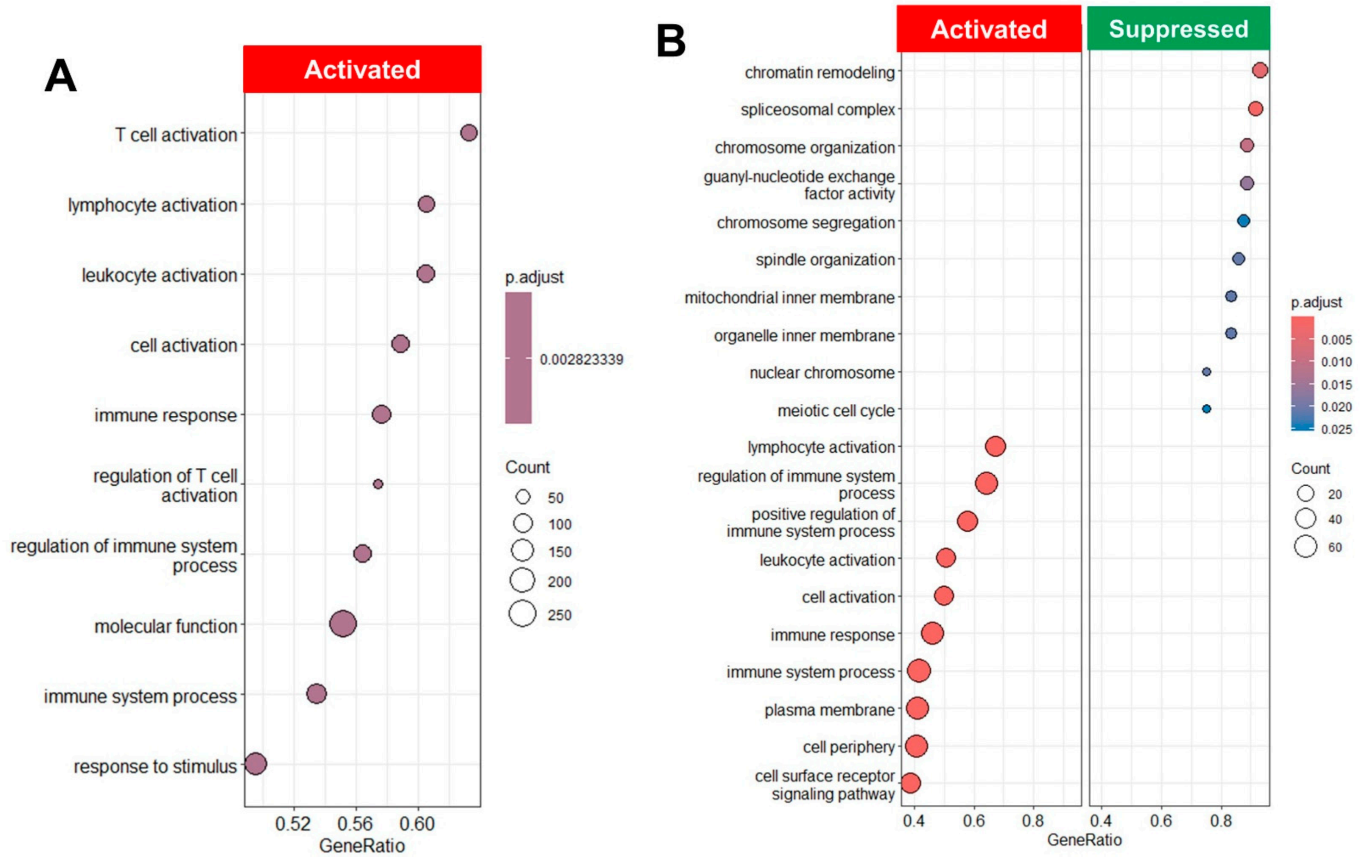

Supplementary Figure S13: Pathway association analysis associated with T cells using GO database.
A. Pathway enrichment analysis using the characteristic genes resulting on the annotation of T cells ($p<10^{-4}$) derived from GSE189955 dataset.
B. Same pathway analysis but resulting from characteristic gene expression from the integrated dataset.
GeneRatio: gene abundance within macrophages versus non-macrophages. *Count*: number of significant genes participating in the GO pathway.
